# Comparing Functional Consequences of Human iPSC‐Microglia and Neural Stem Cell‐Derived Extracellular Vesicles in Mitigating Cognitive Decline in Alzheimer's Disease

**DOI:** 10.1111/acel.70341

**Published:** 2025-12-23

**Authors:** Robert P. Krattli, Mineh Markarian, Shreya Madan, Devyani Swami, Amanda McQuade, Janet E. Baulch, Matthew Blurton‐Jones, Munjal M. Acharya

**Affiliations:** ^1^ Department of Anatomy & Neurobiology University of California Irvine Irvine California USA; ^2^ Department of Neurobiology & Behavior University of California Irvine Irvine California USA; ^3^ Department of Radiation Oncology University of California Irvine Irvine California USA

**Keywords:** Alzheimer's disease, amyloid beta plaques, cognitive function, extracellular vesicles, human induced pluripotent stem cells (iPSC), human microglia, human neural stem cells, microglial activation, neuroinflammation

## Abstract

Stem cell‐derived extracellular vesicles (EVs) show promise as a therapeutic approach for neurodegenerative diseases, particularly Alzheimer's Disease (AD), where traditional regenerative interventions have achieved limited success. Our previous research demonstrated the neuroprotective benefits of human neural stem cell (hNSC)‐derived EVs in 2‐ and 6‐month‐old AD mice (5xFAD) that exibited improved cognitive function and reduced AD‐related neuropathology. This study aimed to compare the neuroprotective efficacy of EVs derived from two human cell lines: hNSCs from H9 embryonic stem cells and human iPSC‐derived microglia (iMGLs). Additionally, we investigated the efficacy of an expanded EV treatment paradigm at subsequently longer time points. Three‐month‐old 5xFAD mice received weekly retro‐orbital vein injections of either hNSC‐ or iMGL‐derived EVs for 4 weeks. Cognitive function testing revealed comparable cognitive improvements in both EV treatment groups compared to vehicle‐injected AD mice. Both iMGL‐ and hNSC‐derived EVs significantly reduced amyloid beta plaques, astrogliosis, and microglial activation, while restoring synaptophysin and postsynaptic density protein PSD‐95 to control levels in AD brains. Gene expression analysis revealed significantly reduced neuroinflammation and elevated neuroprotective signatures following both EV treatments. MicroRNA analysis of the EV‐derived cargo revealed unique and shared miRNA signatures associated with differentially expressed genes in both cell lines. These findings demonstrate the feasibility and neuroprotective benefits of recurrent systemic injections of EVs derived from human NSCs and differentiated human microglia lines in alleviating cognitive dysfunction and neuropathology in Alzheimer's disease.

## Graphical abstract

Systemic (retro‐orbital vein) recurrent injections of human iPSC‐microglia and neural stem cell‐derived extracellular vesicles in a 5xFAD mouse model of Alzheimer's disease significantly improve cortex‐dependent cognitive function, reduce amyloid beta plaques, mitigate microglial and astrocytic activation, deliver neuroprotective miRNA cargo, and improve brain function, demonstrating the feasibility of this regenerative approach.

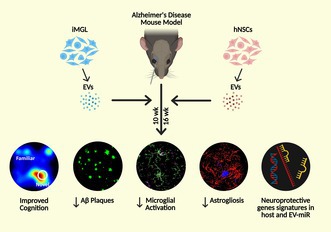

## 1 Introduction

Alzheimer's Disease (AD), one of the most common forms of dementia, affects over 6.7 million Americans aged 65 and older (2023 Alzheimer's Disease Facts and Figures 2023), and is a neurodegenerative disorder marked by progressive cognitive decline and memory loss. AD is characterized by two neuropathological hallmarks: neurofibrillary tangles of hyperphosphorylated microtubule‐associated protein (Tau) and the formation and accumulation of amyloid‐β (Aβ) plaques (Nassrallah et al. 2024). Activated microglia engulf these plaques to remove deposits (Baik et al. 2016). However, elevated microglial activation leads to a pro‐inflammatory state in the AD brain. The upregulation in neuroinflammation and increasing accumulation of Aβ plaques promote neurotoxic events, including neurodegeneration and synaptic loss, which ultimately result in cognitive decline and a reduced quality of life. While there are currently a limited number of therapeutics available to slow this neuropathology, regenerative medicine remains at the forefront of investigation (Felsenstein et al. 2014).

The therapeutic application of neural stem cells (NSCs) in neurodegenerative disorders like AD has shifted from cell replacement to more nuanced approaches such as neurotrophic supplementation (Marsh and Blurton‐Jones 2017). Several AD studies using NSC transplantation in mouse models demonstrated improvements in cognition correlated with improved normal homeostasis via neuroprotective strategies including reduction in proinflammatory glial responses (Lee et al. 2015), improved neuronal metabolic activity (Li et al. 2016), and increased levels of brain derived neurotrophic factor (BDNF) (Blurton‐Jones et al. 2009). However, researchers are moving away from stem cell transplantation in favor of extracellular vesicles (EVs), nano‐scale membrane‐bound organelles that are secreted to mediate intercellular communication among various cell types, and deliver a variety of functional biological cargo such as proteins, mitochondrial components, and microRNA (miR) (Li and Fang 2023). EVs are secreted ubiquitously throughout the extracellular space and promote normal intercellular communication and function (Kumar et al. 2024). Their plasma membrane and expressed surface proteins protect their cargo from degradation and aid in blood brain barrier permeability (Du et al. 2023). A compelling reason for this shift in proposed clinical strategies from cells to cell‐derived EVs is that EV cargo carries similar therapeutic benefits while avoiding certain risks associated with stem cell transplantation, including teratoma formation, host rejection, and invasive surgical delivery (Du et al. 2023; Kumar et al. 2024; Li and Fang 2023; Leavitt et al. 2019). For this reason, more recent AD studies focus on EV treatments that can be administered intravenously (IV) or intranasally (IN) (Li, Liu, et al. 2020; Liu et al. 2020; Losurdo et al. 2020; Leavitt et al. 2019).

In our previous study (Apodaca et al. 2021), we tested the efficacy of hNSC‐EVs via retro‐orbital (RO) vein injections. The RO injection is a rapid systemic delivery method that avoids the loss of therapeutic agents through first‐pass liver metabolism, offers a higher success rate compared to more technically challenging tail vein injections, and is a more humane alternative to other intravenous delivery methods (Yardeni et al. 2011). We observed neuroprotective benefits from RO injections of hNSC‐EVs in that past study; therefore, to remain consistent, we continued using this route of EV delivery in our current study. The 5xFAD model was selected for its rapid neuropathological progression, characterized by Aβ plaque deposition within 2 months and subsequent cognitive dysfunction by 4 months (Padua et al. 2024). Two‐ and 6‐month‐old 5xFAD mice that received one or two doses of hNSC‐EVs, respectively, showed improved cognitive function and reduced AD‐related neuropathology, including plaque accumulation, microglial activation, and synaptic loss. Given the ubiquity of EV communication and the significant contribution of microglial activation to neuroinflammation in the AD brain, it would be prudent to investigate how EVs from other cell types, such as stem cell‐derived microglia, influence disease progression.

In the present study, we compared the therapeutic benefits of hNSC‐derived EVs in 3‐month‐old 5xFAD mice with those of EVs isolated from human induced pluripotent stem cell (iPSC)‐derived microglia (iMGL). Our prior research has shown that iMGL‐derived EVs ameliorated cognitive deficits caused by cytotoxic doxorubicin chemotherapy treatment by attenuating microglial activation and reducing neuroinflammation (Allen et al. 2019). Given the neuroinflammatory and neurotoxic role of microglia in plaque digestion in AD pathophysiology, we tested the hypothesis that homeostatic, non‐diseased human iMGL‐derived EVs could mitigate cognitive deficits and AD neuropathology in a manner and with efficacy similar to hNSC‐derived EVs by decreasing neuroinflammation. We also extended the EV treatment paradigm from one or two intravenous injections to four treatments to expand the therapeutic window of neuroprotective effects.

## 2 Materials and Methods

For detailed methods, protocols, and materials, please see Supporting Information.

### 2.1 Generation, Isolation, and Characterization of hNSC‐ and iMGL‐Derived EVs

The expansion of proliferating human neural stem cells (hNSCs, ENStem‐A line, EMD Millipore) and subsequent media collection were performed according to previously published procedures (Apodaca et al. 2021). Briefly, human iPSC‐derived microglia were differentiated by simplified methods from human iPSC‐derived mesodermal hematopoietic stem cells as described (McQuade et al. 2018). Conditioned media were collected from cultured human iPSC‐derived microglia‐like (iMGL) cells during their differentiation phase (Days 28–35). Extracellular vesicles (EVs) were isolated and purified from conditioned culture medium using ultracentrifugation (Optima XE‐90, Beckman Coulter) as described (Apodaca et al. 2021; Baulch et al. 2016).

EV size and concentration were characterized using nanoparticle tracking analysis (NTA) on a NanoSight NS300 particle analyzer (Laser Spectroscopy Laboratories, UCI). EV morphology was confirmed by transmission electron microscopy (TEM, JEOL 1400 plus, bottom‐mounted with the Gatan OneView camera) at the Cellular and Molecular Medicine Electron Microscopy Core, University of California, San Diego (UCSD‐CMM‐EM Core, RRID: SCR_022039). Further details of the protocols are provided in the Supporting Information section.

### 2.2 Transgenic Mice

All animals used in this study were cared for in accordance with the guidelines provided by NIH and approved by the Institutional Animal Care and Use Committee (IACUC) at the University of California, Irvine. To be consistent with our previous study using hNSC‐EVs in males (Apodaca et al. 2021), we used 12‐week‐old male 5xFAD mice (RRID:MMRRC_034848‐JAX, co‐expressing five familial AD mutations) and their C57Bl/6 age‐matched littermate controls. The mice were housed in standard conditions (20°C ± 1°C; 70% ± 10% humidity; 12:12 h light/dark cycle) in groups of 2–4 per cage. The 5xFAD mouse model was selected for its early onset of AD neuropathology, specifically the deposition of amyloid‐β (Aβ) plaques, synaptic loss, and cognitive impairments.

### 2.3 EV Administration

5xFAD mice and WT controls were divided into the following groups: vehicle‐injected AD with hibernation buffer (Gibco; AD; *N* = 12), hNSCs‐EV‐injected AD (AD + hNSC‐EV; *N* = 14), iMGL‐EV‐injected AD (AD + iMGL‐EV; *N* = 14), and vehicle‐injected wild type (WT, *N* = 16). Mice were 12–13 weeks old at the start of EV treatment, which was administered via retro‐orbital (RO) vein injection. Our previous studies have shown that either intra‐hippocampal stereotaxic surgery or injection into the RO sinus provides equivalent efficacy (Apodaca et al. 2021; Leavitt et al. 2020). RO injections (EV or hibernation buffer vehicle) were administered once a week for 4 weeks, with an EV dose of 2.25 × 10^7^ EVs/50 μL per injection.

### 2.4 Cognitive Function Testing

Behavioral testing was administered 1 month after the final EV treatment. Testing was conducted over 2 weeks and included the following tasks: open field testing (OFT) in conjunction with object recognition memory (ORM), elevated plus maze (EPM), and fear extinction (FE) memory. All behavior videos were scored by independent investigators who were blinded to the experimental groups. OFT, ORM, and EPM were scored manually, while FE was scored using FreezeFrame (Coulbourn Instruments). Detailed test protocols are available in the Supporting Information S1 section.

OFT was conducted on the first day of habituation for the ORM test. In OFT, avoidance of the inner 60% square area of the total arena indicates neophobic, anxiety‐like behavior, while active exploration implies the opposite. The ORM task assesses episodic memory by measuring the animal's preference for a novel object over a familiar one. A Memory Index was calculated for each mouse using the formula: [(novel exploration time/total exploration time) − (familiar exploration time/total exploration time)] × 100. EPM also measures anxiety levels; the maze is constructed of two bisecting platforms—one enclosed and preferred by anxious animals, and the other open, shaped like a plus sign. Time spent in the open versus closed arms was measured and compared among groups to evaluate anxiety‐like behavior. The FE memory task assesses amygdala‐hippocampal‐cortical circuit‐dependent fear conditioning learning and memory consolidation. This 5‐day task includes three phases: conditioning, extinction training, and the fear test. On Day 1, animals experienced three pairings of an auditory tone followed by a mild foot shock. Twenty‐four hours later, the mice underwent 3 days (Days 2–4) of exposure to 20 tones without shocks in the same fear chamber. On Day 5, the fear test was conducted, during which three tones were played in the same context without foot shocks. The percentage of time each animal spent freezing was calculated for all phases. Freezing times were averaged across 5‐tone intervals for a total of 4 data points for each day of the extinction training phase (Days 2–4).

### 2.5 Fluorescence Staining

Mice were anesthetized with isoflurane and euthanized via intracardiac perfusion with 4% paraformaldehyde (PFA) in phosphate buffered saline (PBS) (100 mM, pH 7.4, Sigma). Brains were cryoprotected using a 10%–30% sucrose gradient and cryo‐sectioned (HN525 NX, Epredia, Germany) at a coronal thickness of 30 μm. For each fluorescence stain, sections were collected from each group at two distinct time points (10 weeks and 16 weeks post‐EV injections) and from 2 brain regions: medial prefrontal cortex (mPFC) and perirhinal cortex (PRh).

We used Thioflavin S (0.5% solution, Sigma) to quantify Aβ plaque deposition in an ethanol gradient wash. Synaptic integrity was measured using the presynaptic protein synaptophysin (1:1000, Synaptic Systems) and the post‐synaptic density protein 95 (PSD‐95, 1:1000, Invitrogen). To observe the plaque‐targeted immune response, we utilized a fluorescent plaque stain, AmyloGlo (Biosensis), in conjunction with two glial cell markers: the disease‐associated microglia (DAM) protein CD9 (1:150, Biolegend) and glial fibrillary acidic protein (GFAP 1:500, Sigma). Fluorescence was visualized using goat anti‐mouse Alexa Fluor 568 (1:500). GFAP^+^ astrocytic activation was also quantified via C3d immunoreactivity (1:250, R&D Systems). To label the activated microglia, tissues underwent dual immunofluorescence staining with a pan microglial marker (IBA1) and a lysosomal protein, CD68 (rabbit anti‐IBA1, 1:500, Wako; rat anti‐mouse CD68, 1:500, BioRad). Fluorescence was visualized using goat anti‐rat Alexa Fluor 647 (1:1000) and goat anti‐rabbit Alexa Fluor 488 (1:500) secondary antibodies. Detailed staining protocols are available in the Supporting Information section.

All immunofluorescence‐stained sections were imaged using a Nikon Eclipse AX laser scanning microscope (Nikon Eclipse AX, Japan) equipped with either a 20× air or 40× oil immersion objective lens. Volumetric z stacks were used to construct *in silico* 3D surface models to quantify the volume of immunoreactivity for each cellular marker (ClearView, Imaris v10, Andor Technologies). Aβ plaque counts were performed manually using the Imaris‐built surfaces and normalized to the vehicle‐treated AD group.

### 2.6 Transcriptomic Profile of Neuroinflammation‐Related Genes

To determine the therapeutic effects of hNSC‐ and iMGL‐derived EV treatments on AD brain neuroinflammation gene expression, samples from 18 mice (*N* = 3–4 mice per group), from the Early Time Point (10 weeks post‐EV treatment), were analyzed using the nCounter Mouse Neuroinflammation Panel (NanoString). Sample processing and data collection were performed by the Genomics Research and Technology Hub at UC Irvine. The raw value data were normalized on the nCounter module utilizing a two‐step normalization process: an initial Positive Control Normalization, followed by a CodeSet Content Normalization using 13 housekeeping genes.

### 2.7 RNA Sequencing of Unique EV miRNA Cargo

MicroRNAs (miR) are key bioactive cargo within EVs that exert varied and long‐reaching effects on intra‐ and inter‐neuronal activity (Leavitt et al. 2020, 2019). To better characterize and compare the unique miR signatures of hNSC‐ and iMGL‐derived EVs, RNA was isolated and purified from EV stock solutions using the RNEasy Kit (Qiagen); nucleic acid purity was verified with both NanoDrop (ThermoFisher) and Bioanalyzer 2100 (Agilent Technologies) instruments. Isolated RNA from hNSC‐derived EVs was processed and analyzed in duplicate on a miR microarray chip (Exiqon, Denmark; Genomics Shared Resource at the University of Texas Southwestern Medical sCenter) as previously described (Apodaca et al. 2021). iMGL‐derived EVs were sequenced using the NEXTFLEX Small RNA Sequencing Kit v4 (Revvity). The Genomics Research and Technology Hub at UC Irvine conducted processing and data organization. Functional enrichment analysis of predicted miRNAs derived from EV‐iMGLs and hNSC‐EV samples was conducted using Metascape, based on the predicted target genes associated with each miRNA. Enriched biological processes and pathways were identified using default Metascape parameters.

### 2.8 Cytokine Expression Analysis

Freshly dissected hippocampi from each brain (*N* = 4 per group) were homogenized, washed, and supernatants collected for the multiplex cytokines analysis using Q‐Plex 14 cytokine array kit to conduct an enzyme‐linked immunosorbent assay (ELISA).

### 2.9 Statistical Analysis

All data are expressed as the mean ± SEM. Statistical analyses of cognitive function, biochemical, and immunohistochemical data were conducted using two‐way ANOVA (GraphPad Prism, v8.0). For the analysis of AD, hNSC‐ or iMGL‐derived EV effects, two‐way ANOVA and Bonferroni's multiple comparisons tests were applied. The exploration of familiar versus novel objects in the ORM task by the same animals was compared using the Wilcoxon matched‐pairs signed‐rank test. Fear extinction training data were analyzed using repeated measures ANOVA and Bonferroni's multiple comparisons tests. All statistical analyses were considered significant for a value of *p* ≤ 0.05.

## 3 Results

### 3.1 hNSC‐ and iMGL‐Derived EVs Ameliorate Cognitive Impairment in 5xFAD Mice

**FIGURE 1:**
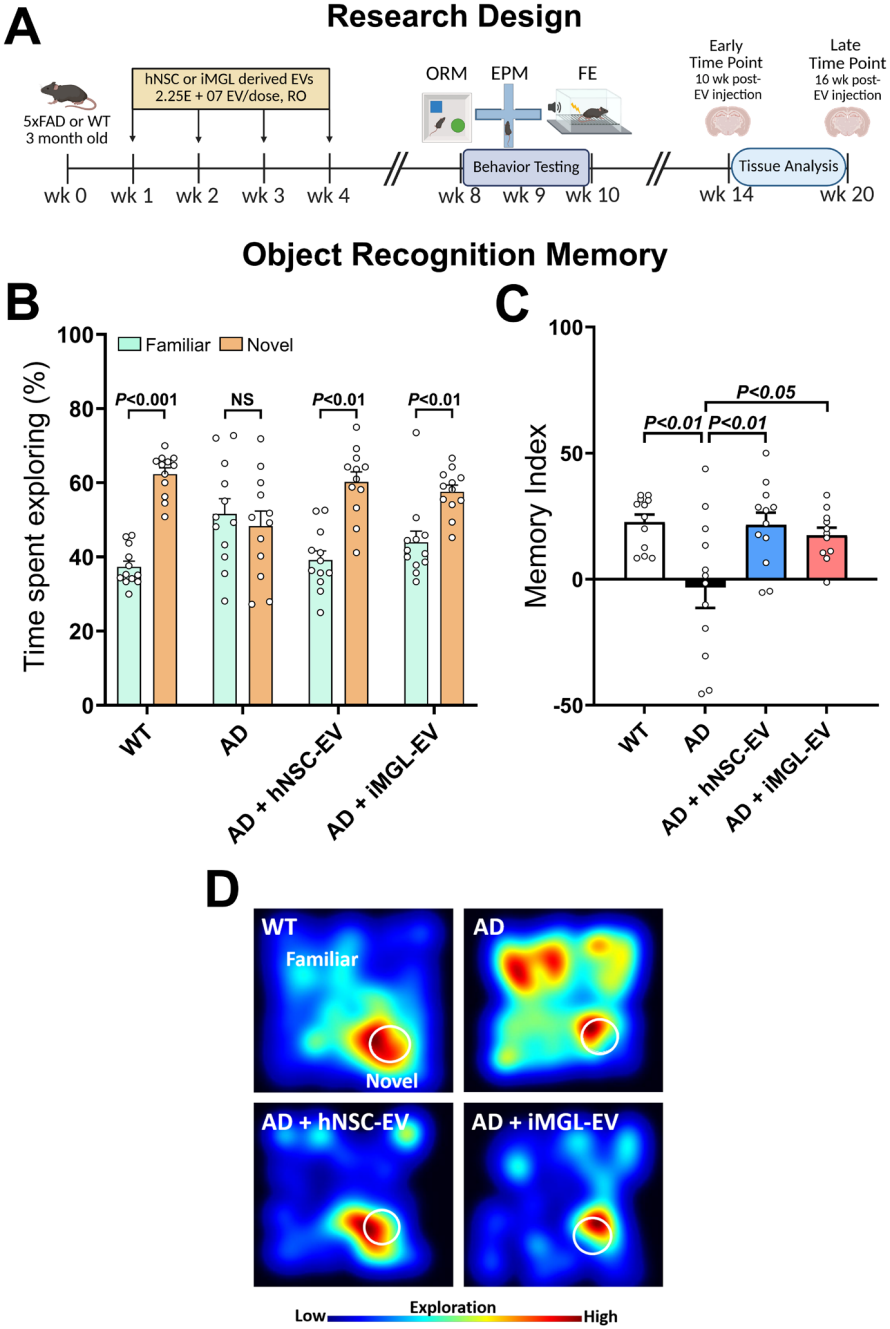
iMGL and hNSC derived EVs protect against Alzheimer's Disease‐related cognitive impairment. (A) Experimental research design: 3‐month‐old male 5xFAD mice were administered 4 weekly treatments of either vehicle, iMGL‐ or hNSC‐derived extracellular vesicles (2.25 × 10^7^ EVs/50 μL injection volume) via retro‐orbital vein injection. Age‐matched C57Bl/6 WT mice injected with vehicle (hibernation buffer) served as controls. One month after the last RO injection, all cohorts of animals were administered cognitive function tests, including Object Recognition Memory (ORM), Elevated Plus Maze (EPM), and Fear Extinction Memory Consolidation (FE) as described in the Supporting Information. Animals were then euthanized for tissue collection at either 10 or 16 weeks after their last EV treatment, corresponding to an animal age of 30 or 36 weeks, respectively, and referred to as “early” and “late” analysis intervals. (B) Quantification of percentage time spent exploring novel and familiar objects during test phase of ORM. The vehicle‐treated AD group showed no significant difference between the percentage of time exploring the novel versus the familiar object. In contrast, WT, AD + hNSC‐EV, and AD + iMGL‐EV showed elevated novel object exploration. (C) Memory performance during ORM was quantified as a Memory Index (MI), calculated by dividing the difference in time explored for the novel and familiar object by the total time explored for both objects. Vehicle‐treated AD mice showed significantly reduced MI scores, while AD mice treated with either hNSC or iMGL‐EVs showed restoration of MI that was comparable to the WT control group. (D) Representative heatmaps of object exploration during the ORM behavior test. Heatmaps for both hNSC‐ and iMGL‐EV‐treated AD groups displayed a higher exploratory preference for the novel in comparison with familiar object comparable to the WT mice. In contrast, the untreated AD group lacked preference for the novel object. Data are presented as mean ± SEM (*N* = 12–16 mice/group), ANOVA, and Bonferroni's multiple comparison test.

EV morphology was characterized using transmission electron microscopy (TEM, Figure S1). The average size of hNSC EVs was 115.0 ± 0.4 nm in diameter with a mean stock concentration of 7.90 × 10^8^ ± 6.58 × 10^6^ per mL (Figure S1A,B). iMGL‐EVs were 118.6 ± 1.1 nm in diameter at a concentration of 1.01 × 10^9^ ± 2.67 × 10^7^ per mL (Figure S1C,D). EVs were then diluted using hibernation buffer (Gibco) for RO vein injections. Adult male 5xFAD mice received four weekly RO injections of either vehicle, hNSC‐ or iMGL‐derived EVs at a concentration of 2.25 × 10^7^ EVs per 50 μL treatment dose; age‐matched C57BL/6 male mice served as controls (Figure 1A). At 1 month post‐treatment, mice were handled and then administered cognitive function tasks to determine the therapeutic efficacy of EVs. We assessed hippocampus‐ and perirhinal cortex‐dependent learning and memory using the object recognition memory (ORM) task. The percentage of time spent exploring the novel object was higher compared to the familiar object in WT control mice, as well as in both hNSC‐ and iMGL‐EV treated AD mice (*p* < 0.001, 0.01, and 0.01, respectively); there was no significant difference in object exploration time by vehicle‐treated AD mice (Figure 1B). The observed exploration times were then utilized to calculate a memory index (MI), where positive values indicate novel object preference (Figure 1C). We found significant differences between experiment groups (Figure 1D, *F*_(3,43)_ = 5.494, *p* < 0.003). This difference in object exploration, or the lack of it, was also evident when visualized as a heatmap of summarized animal exploration activity (Figure 1D). Vehicle‐treated AD mice demonstrated a significantly reduced MI score when compared to WT control mice, and as compared to both hNSC‐ and iMGL‐EV‐treated AD mice (*p* < 0.01, 0.01, and 0.05, respectively). Importantly, both hNSC‐ and iMGL‐EV‐treated AD mice showed no significant difference in MI score when compared to WT control.

We observed a lack of intergroup variation during the Open Field Test (OFT) conducted on the first day of ORM to assess neophobic behavior. There was no statistically significant difference in time spent in the central zone, defined as the inner 60% of the ORM arena field (Figure S2A). To test for anxiety‐like behavior, mice underwent the elevated plus maze (EPM) test, conducted under bright overhead lighting and consisting of intersecting open and closed walkways. Statistical analysis showed no significant difference in the percentage of exploration time spent in the open arms across any of the experimental groups (Figure S2B). These results indicate that neither AD pathology nor EV treatment affects anxiety levels and spontaneous‐based exploration cognitive testing.

We also investigated the effects of EV treatment on learning and memory in the amygdala‐cortical‐hippocampal circuit via a 5‐day contextual‐based fear memory and consolidation task (Figure S2C,D). All four experimental groups exhibited a significant and comparable increase in time spent freezing during the tone‐shock conditioning phase on Day 1 (Figure S2C; T_1_–T_3_; 45%–55% on T_3_), with neither vehicle‐treated nor EV‐treated AD mice affected in their acquisition of the conditioned fear response. During the extinction phase (Days 1–3, D1‐D3), we observed an overall decrease in freezing time across all four groups during Days 1 and 2. Compared to vehicle AD, both WT and hNSC‐EV‐treated mice spent more time freezing during Days 1 and 2 (*p* < 0.01), a phenomenon not observed on Day 3. There was no significant difference between groups in the time spent freezing during the extinction test on Day 5 (Figure S2D).

### 3.2 hNSC‐ and iMGL‐Derived EVs Reduce Beta‐Amyloid Plaque Deposition

**FIGURE 2:**
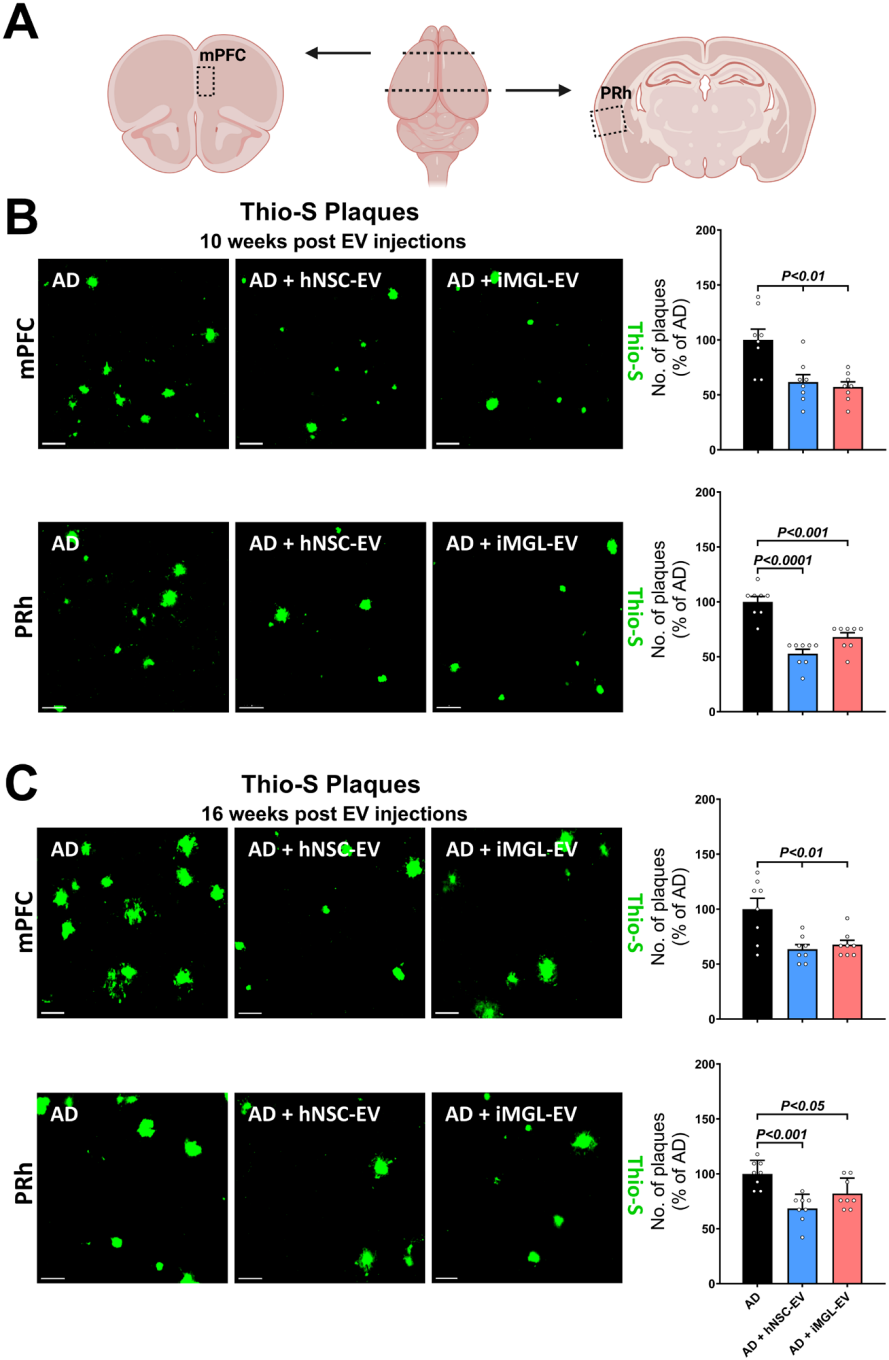
iMGL and hNSC‐derived EVs reduce beta‐amyloid plaque deposition at the early and late intervals in 5xFAD mice cortices. (A) Anatomical visualization of mouse brain regions of interest pertinent to behavior outcomes and immunohistochemistry staining: the medial prefrontal cortex (mPFC) and perirhinal cortex (PRh). (B) Representative confocal micrographs and quantification of the number of beta‐amyloid plaques in the mPFC and PRh regions at the early time interval (10 weeks) after the last EV injection. Tissues were stained with the Thioflavin‐S (Thio‐S), and images were acquired via laser scanning confocal microscopy. Image analysis showed a reduction in plaque number in both hNSC‐ and iMGL‐EV‐treated AD groups compared with vehicle‐treated AD mice. Plaque accumulation was significantly reduced in both regions of interest. (C) Quantification of the Thio‐S‐stained plaques for the late time interval of 16 weeks post EV treatment. Fluorescence imaging and plaque quantification showed that hNSC‐ and iMGL‐ derived EVs significantly reduced plaque deposition in the mPFC compared to the vehicle‐ treated AD. Plaque count was also significantly decreased for both EV‐treated groups in the PRh region. Data are presented as mean ± SEM (*N* = 4 mice/group), ANOVA, and Bonferroni's multiple comparison test. Scale bar, 40 μm (B, C).

To determine the effects of EV treatment on AD neuropathology, we quantified Aβ plaque accumulation, a hallmark of the 5xFAD mouse model, in the medial prefrontal cortex (mPFC) and perirhinal cortex (PRh) of the vehicle, hNSC‐ and iMGL‐EV‐treated AD mice (Figure 2A). Plaque counts were acquired via Thio‐S fluorescence staining at both 10 weeks (Figure 2B) and 16 weeks (Figure 2C) post‐EV treatment to discern any long‐term efficacy of EV treatment. At 10 weeks post‐treatment, both hNSC‐ and iMGL‐EV‐treated AD mice showed a significant decrease in the number of Thio‐S^+^ plaques in both the mPFC (*p* < 0.01 and 0.01, respectively) and PRh (*p* < 0.0001 and 0.001, respectively) compared to vehicle‐treated AD mice. Analysis of the plaque counts at 16 weeks post‐EV treatment revealed similar results where mice treated with hNSC‐ and iMGL‐derived EVs showed significantly fewer Aβ plaques in both the mPFC (*p* < 0.01 and 0.01, respectively) and PRh (*p* < 0.001 and 0.05, respectively) regions of the brain as compared to vehicle‐treated AD mice. The reduction observed at the late time point indicates that both hNSC‐ and iMGL‐derived EVs exert prolonged protection against plaque load in the 5xFAD mouse brain.

### 3.3 hNSC‐ and iMGL‐Derived EVs Protect Against AD‐Related Synaptic Loss

**FIGURE 3:**
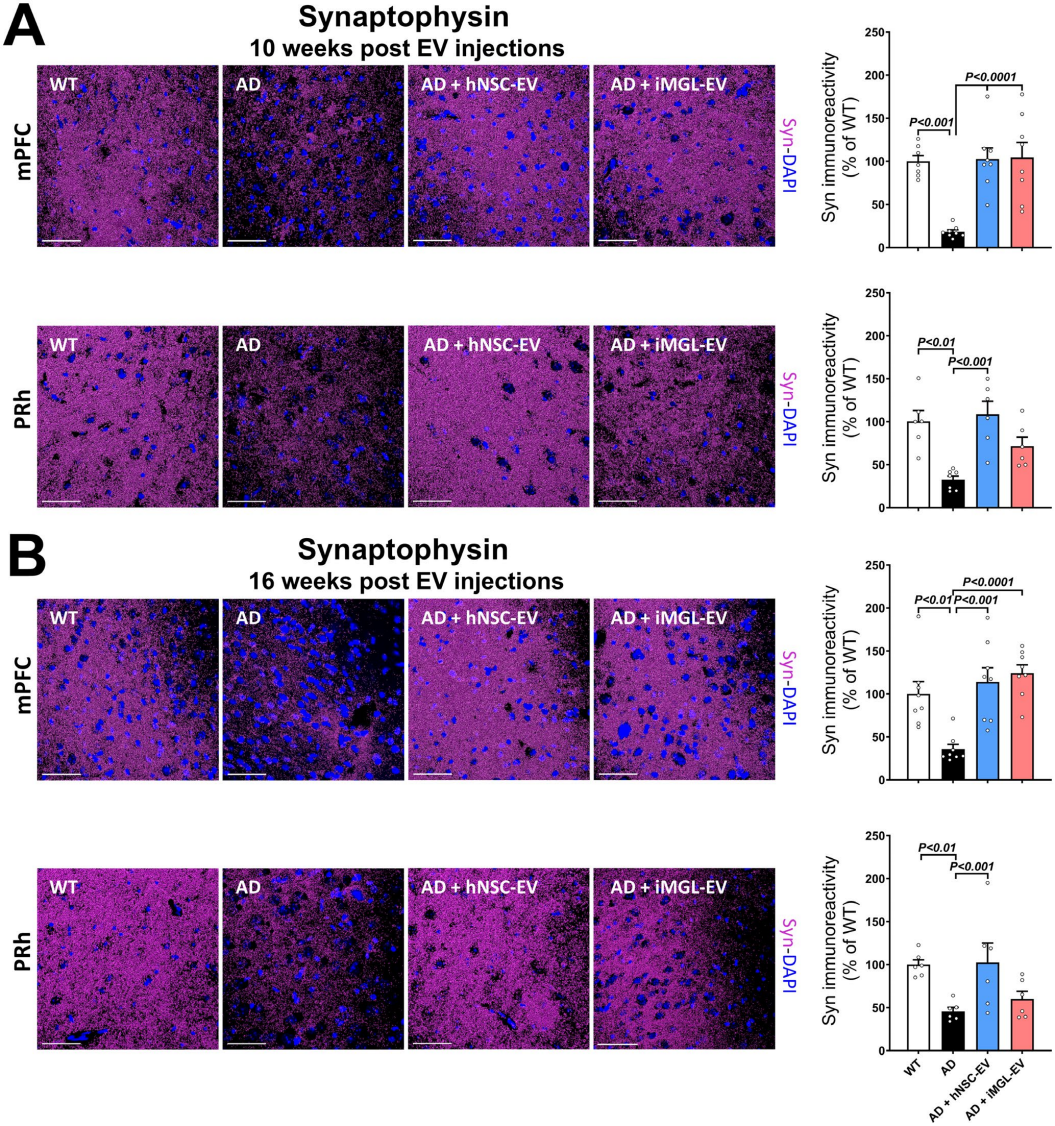
hNSC and iMGL derived EVs prevent cortical synaptic loss in Alzheimer's disease mice brains. (A) Representative images and quantification of synaptophysin (Syn) immunoreactivity (magenta, synaptophysin; blue, DAPI nuclear counterstain) for the early time interval (at 10 weeks) after the last EV treatment. Immunofluorescence staining, laser scanning confocal microscopy, and volumetric image analysis were performed in the medial prefrontal (mPFC) and perirhinal (PRh) cortices. (B) Quantification of synaptophysin for the late time interval cohort at 16 weeks post EV treatment. Data are presented as mean ± SEM (*N* = 4 mice/group), ANOVA, and Bonferroni's multiple comparison test. Scale bar, 50 μm (A, B).

Degeneration of neuronal synapses strongly correlates with cognitive decline observed in AD (Meftah and Gan 2023). Therefore, to determine whether hNSC‐ and iMGL‐derived EVs exert an effect on synaptic integrity in the 5xFAD mouse, we evaluated the immunoreactivity of the postsynaptic marker synaptophysin (Syn) in the mPFC and PRh cortical regions of WT control mice and AD mice treated with vehicle, hNSC‐, or iMGL‐EVs. At 10 weeks post‐treatment, results showed a significant decrease in Syn expression in both the mPFC and PRh regions of vehicle‐treated AD mice compared to WT controls (Figure 3A; *p <* 0.001 and 0.01, respectively). In contrast, AD mice treated with hNSC‐derived EVs exhibited higher Syn protein levels in both mPFC and PRh regions at 10 weeks post‐EV treatment compared to the vehicle‐treated AD group and similar to WT controls (*p* < 0.0001 and 0.001, respectively). iMGL‐EV‐treated AD mice from the early time point exhibited a significant increase in Syn immunoreactivity in the mPFC region compared to the vehicle‐treated AD group (*p* < 0.0001); a similar trend was observed in the PRh. At 16 weeks post‐treatment, vehicle‐treated AD mice also demonstrated a significant decrease in Syn in both the mPFC and PRh regions compared to the WT controls (Figure 3B; *p* < 0.01). AD mice treated with hNSC‐derived EVs showed a significant increase in Syn protein levels compared to the vehicle‐treated AD group in the mPFC and the PRh (*p* < 0.001). iMGL‐derived EVs also exerted a significant effect on Syn expression in the mPFC (*p* < 0.0001) when compared to vehicle‐treated AD mice that was similar to WT controls. A trend for increased synaptic protection that did not reach statistical significance was seen in the PRh. To assess the impact of EV treatment on post‐synaptic density, we performed PSD‐95 staining in the mPFC at 10 weeks post‐EV injection (Figure S3). PSD‐95 was significantly reduced in the AD mPFC compared to WT controls (*p* < 0.003), and treatment with hNSC‐EV significantly increased PSD‐ 95 (*p* < 0.03). In contrast, the mPFC treated with iMGL‐EV showed a trend toward increased PSD‐95 immunoreactivity that did not reach statistical significance compared to the AD‐Vehicle group.

### 3.4 hNSC‐ and iMGL‐Derived EVs Decrease Proinflammatory Glial Responses in AD Brain

**FIGURE 4:**
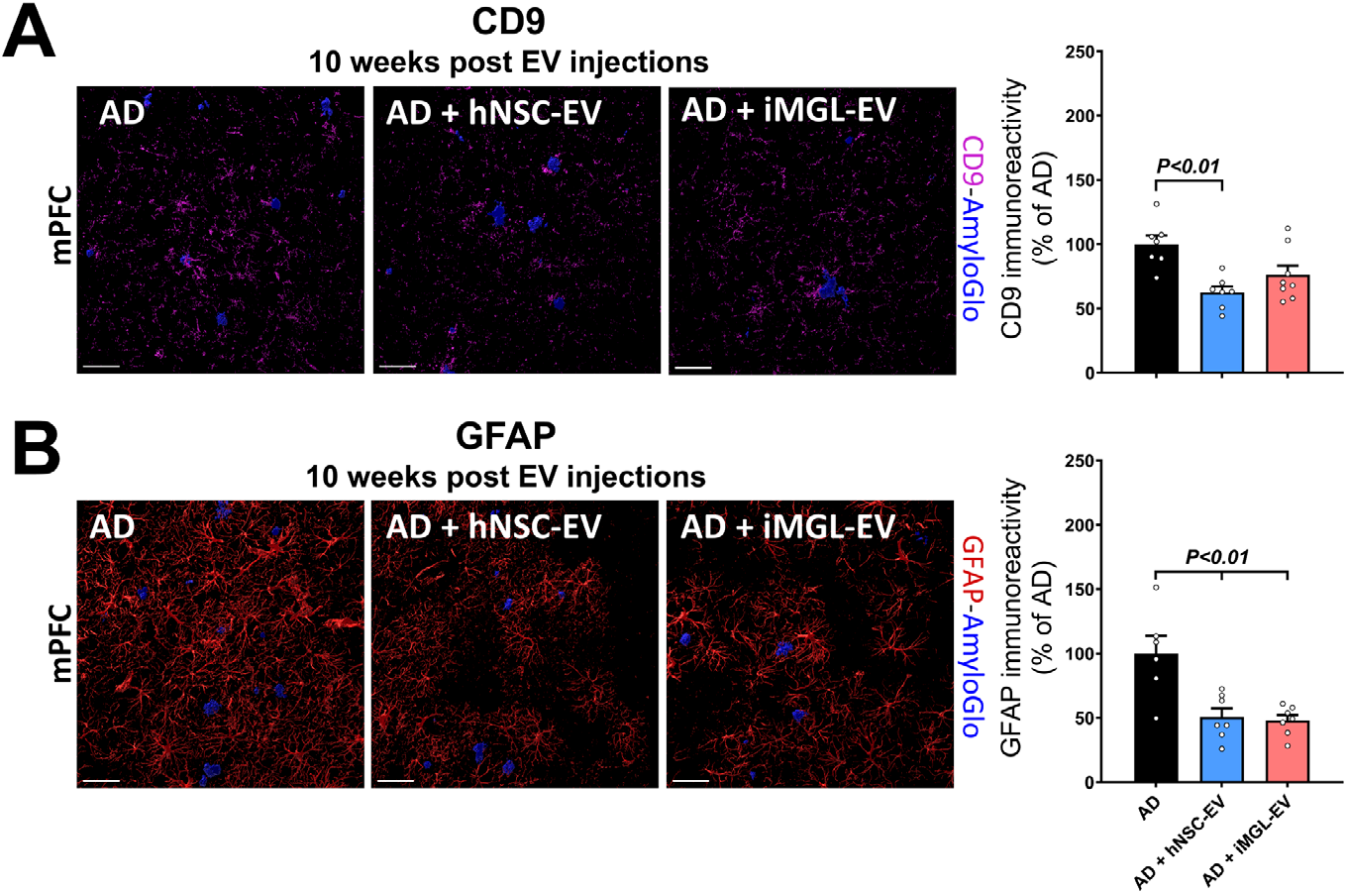
hNSC and iMGL derived EVs alleviate reactive glial response to beta‐amyloid plaques in medial prefrontal cortex. (A) Visual representation and quantification of disease associated microglia (DAM, CD9, magenta) in response to beta‐amyloid plaque deposits (Amylo‐Glo, blue). Confocal micrographs of the medial prefrontal cortex (mPFC) at the early time interval (10‐weeks post EV‐treatment) are shown for the AD + Vehicle, AD + hNSC‐ EV, and AD + iMGL‐EV groups. 3D algorithm‐based volumetric quantification (Imaris) showed reduced CD9 immunoreactivity in the AD brain following hNSC‐EV treatment compared to vehicle‐treated AD mice. (B) Volumetric analysis of astrogliosis (GFAP^+^, red) demonstrated a significant decrease in GFAP^+^ astrocytic processes surrounding plaques (Amylo‐Glo, blue) in the mPFC region of AD mice following treatment with EVs derived from hNSC and iMGL compared to the vehicle‐treated AD mice. Data are presented as mean ± SEM (*N* = 4 mice/group), ANOVA, and Bonferroni's multiple comparison test. Scale bar, 40 μm (A, B).

The recruitment and activation of microglia and astrocytes in the AD results in a positive feedback loop that leads to a chronically neurotoxic microenvironment (Kim et al. 2018). At 10 weeks post‐EV treatment we investigated whether hNSC‐ and iMGL‐derived EVs affect this microenvironment by analyzing the expression of glial activation markers, including: CD9, expressed by disease‐associated microglia protein (DAM) and glial fibrillary acidic protein (GFAP), as markers of astrocytes and astrogliosis (Figure 4). Volumetric quantification revealed a significant decrease in CD9 immunoreactivity in the mPFC region of hNSC‐EV‐treated AD mice at 10 weeks post‐EV treatment compared to the vehicle‐treated AD group (Figure 4A; *p* < 0.01). A comparable trend was observed in the iMGL‐EV‐treated mice. We did not find any significant difference in CD9 expression levels between the three AD groups in the PRh regions at 10 weeks, nor in either brain region at 16 weeks post‐treatment (Figure S4A–C). A similar pattern was observed in the analysis of GFAP immunoreactivity. At 10 weeks post EV‐treatment, GFAP expression levels were significantly decreased in the mPFC region of both hNSC‐ and iMGL‐EV‐treated AD mice compared to the vehicle‐treated AD group (Figure 4B; *p* < 0.01), but not for the PRh region (Figure S4D). There were also no significant differences in GFAP immunoreactivity in either brain region at the later time point (Figure S4E,F). Concurrently, dual immunofluorescence and colocalization analysis of a complement anaphylatoxin component C3d with astrocytes (GFAP^+^, Figure S5A–C) showed a significant reduction in C3d‐GFAP colocalization in the AD brain treated with hNSC‐ and iMGL‐EV (mPFCs) compared to the vehicle‐treated AD group at 10 weeks post‐EV injections, indicating reduced astrocytic activation.

**FIGURE 5:**
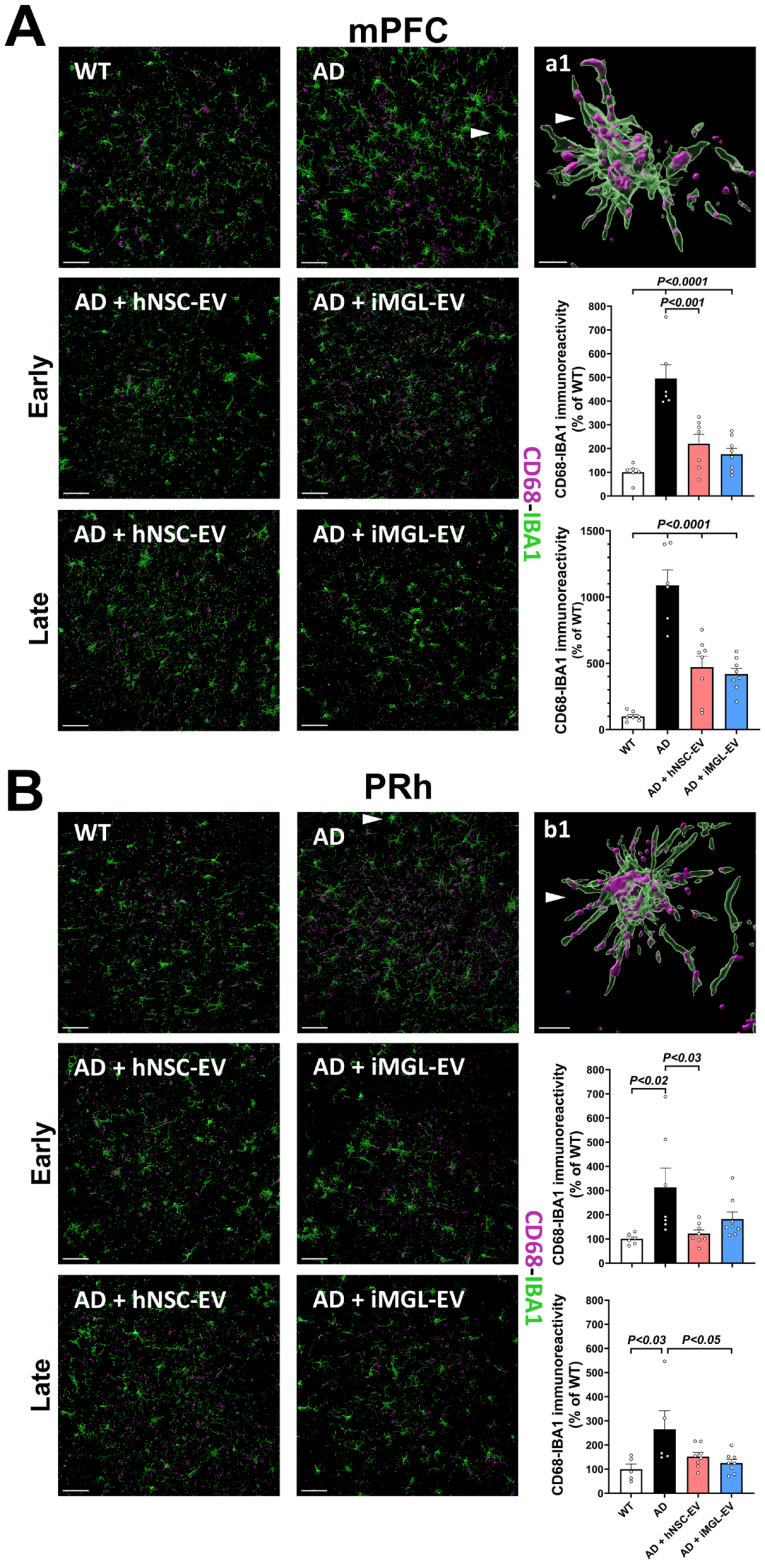
hNSC and iMGL derived EVs temper phagocytic microglial activity in AD cortex. (A) Representative images and volumetric analysis of activated microglia in the mPFC regions of experimental groups from the early and late time intervals (10 weeks and 16 weeks post EV treatment, respectively). Microglial activation was quantified via the colocalized immunofluorescent expression of a lysosomal membrane protein (CD68, magenta), and a pan‐ microglial calcium binding adapter protein (IBA1, green). Vehicle‐treated AD mice showed a significant increase in CD68‐IBA1 co‐labeled immunoreactivity at the early and late time points compared to WT controls; a1 depicts a magnified view of an activated microglial cell from the vehicle‐treated AD group (white arrow), shown as a 3D surface rendering. AD mice treated with both hNSC‐ and iMGL‐derived EVs showed a significant decrease in CD68‐IBA1 colocalization in the mPFC at both the early and late time intervals. (B) Quantification and visual representation of colocalized CD68‐IBA1 expression in the PRh region of the early and late time interval experimental groups. A significant increase in CD68‐IBA1 immunoreactivity was observed in the vehicle‐treated AD mice at both time points, which was significantly reduced in the hNSC‐EV‐treated AD mice from the early time interval and in the iMGL‐EV‐treated AD mice from the late time interval; a similar trend in reduction was observed for the other brain regions and post‐treatment intervals. A 3D volumetric surface of a single activated microglial cell from the vehicle AD group (white arrow) is represented in b1. Data are presented as mean ± SEM (*N* = 4 mice/group), ANOVA, and Bonferroni's multiple comparison test. Scale bar, 50 μm (A, B) and 5 μm (a1, b1).

To assess the impact of EV treatment on the phagocytic profile of activated microglia in the AD brain, we quantified the expression of lysosomal protein CD68 in conjunction with the pan‐microglial marker IBA1 (ionized calcium‐binding adaptor molecule) using dual immunofluorescence staining and 3D algorithm‐based volumetric quantification of co‐localized, immunoreactive surfaces. We observed a significant increase in the co‐labeled immunoreactivity of CD68‐IBA1 in the mPFC region of AD mice at both 10 and 16 weeks compared to the WT control group (*p* < 0.0001, Figure 5A,a1) while treatment with both hNSC‐ and iMGL‐derived EVs significantly reduced microglial activation in the mPFC region of 5xFAD mice compared to vehicle‐treated AD mice at the 10 week post‐treatment time (*p* < 0.001 and 0.0001, respectively). CD68‐IBA1 colocalization was also significantly reduced in the mPFC of both hNSC‐EV‐treated and iMGL‐EV‐treated AD mice 16 weeks post‐EV treatment (*p* < 0.0001). Compared to the WT control, CD68‐IBA1 immunoreactivity was significantly elevated in the PRh region of vehicle‐treated AD mice at both 10 and 16 weeks (*p* < 0.02 and 0.03, respectively; Figure 5B,b1). Microglial activation was significantly reduced in the hNSC‐EV‐treated AD group at 10 weeks post‐EV treatment (*p* < 0.03), with a similar trend observed in AD mice treated with iMGL‐derived EVs. At 16 weeks post‐EV treatment, reduced CD68‐IBA1 colocalization was found in the PRh of the iMGL‐EV‐treated AD group (*p* < 0.05).

### 3.5 hNSC‐ and iMGL‐Derived EVs Uniquely Impact Transcriptome in the 5xFAD Brain

**FIGURE 6:**
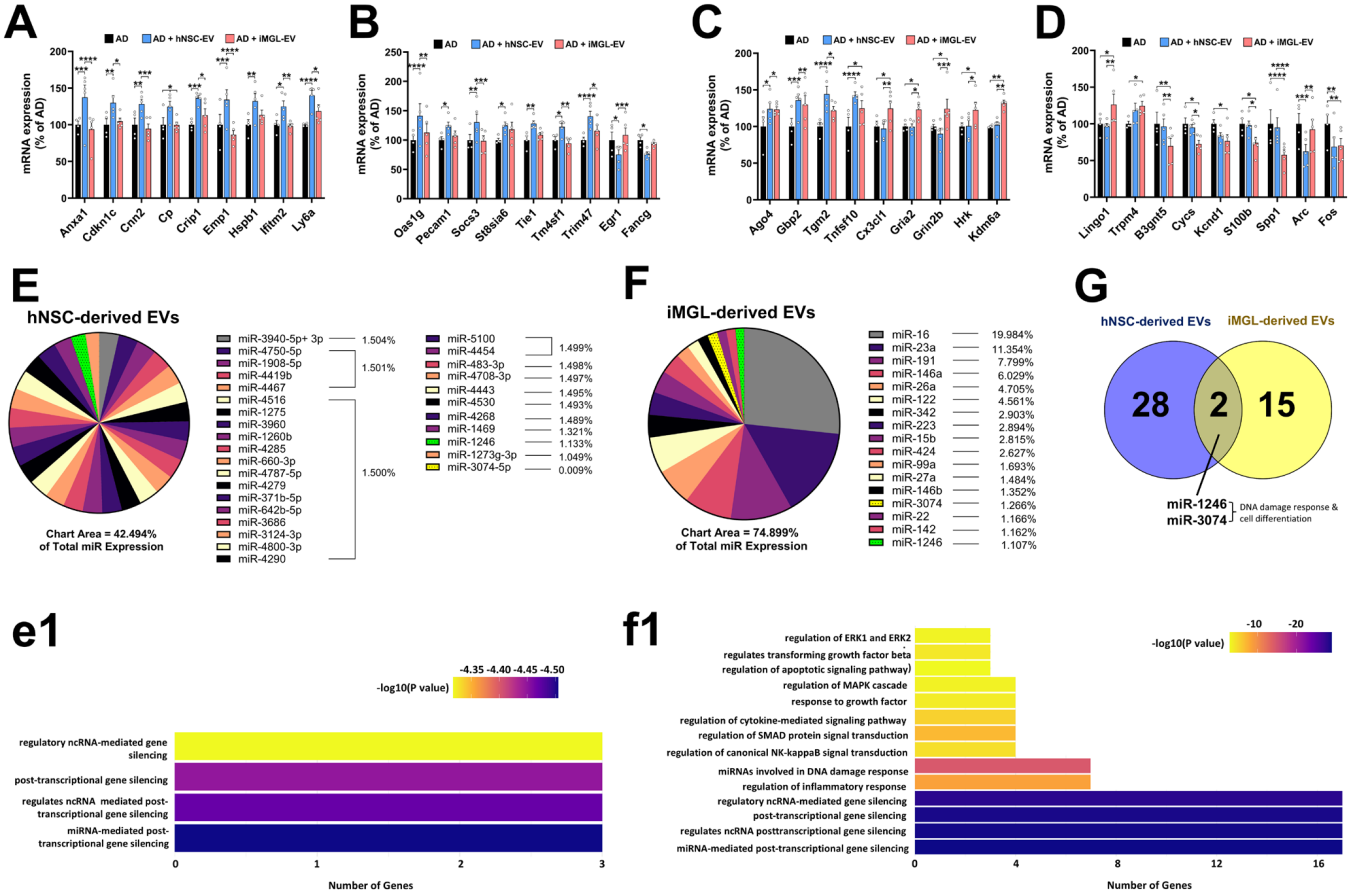
Unique miRNAs within hNSC‐ and iMGL‐derived EV cargo produce differences in transcriptomic profiles in the host mouse AD brain. (A–D) EV‐induced gene expression changes in 5xFAD mouse brains treated with vehicle (black), hNSC‐ (blue), or iMGL‐ (red) derived extracellular vesicles. RNA‐seq analysis of a 757‐gene neuroinflammatory panel (NanoString) yielded 48 hits whose log2 fold changes in mRNA expression met selection criteria (*p* < 0.05). Normalized mRNA counts for the selected genes in the hNSC‐EV and iMGL‐EV treatment are presented as a percentage of AD + Vehicle group (A–D). Data are presented as mean ± SEM (*N* = 4–5 mice/group), ANOVA, and Bonferroni's multiple comparison test. (*p < 0.05, **p < 0.01, ***p < 0.001, ****p < 0.0001). (E, F) Quantification of microRNA (miR) contained within hNSC‐ (E) and iMGL‐derived (F) EV cargo. Small RNA derived from both EV sources underwent RNA‐seq analysis to generate unique miR library sets. Selection criteria included miRs with gene counts constituting more than 1% of total miR expression or those expressed in both library sets. The pie chart area represents the percentage of the most highly expressed miRs. (e1 and f1) Functional enrichment pathway analysis of predicted miRNAs from hNSC‐ (e1) and iMGL‐derived (f1) EVs was performed using Metascape based on their predicted target genes. The *X*‐axis represents the number of genes from the EV‐iMGLS dataset associated with each enriched pathway, and the *Y*‐axis lists the corresponding biological processes or pathways identified through enrichment analysis. The color scale indicates the statistical significance of enrichment, expressed as −log10 (*p*‐value). (G) Venn diagram showing the number of unique and shared miRs between hNSC‐ and iMGL‐derived EVs. hNSC‐derived EVs yielded 28 unique miRs, while iMGL‐derived EVs yielded 15 uniquely expressed miRs. Two miR species, miR‐1246 and miR‐3074, were expressed in both EV sources.

Changes in the transcriptomic profiles of 5xFAD mice receiving either vehicle, hNSC‐ or iMGL‐derived EVs were analyzed using RNA‐sequencing data obtained from the commercially available nCounter Mouse Neuroinflammation Panel (NanoString). Analysis of the 757 gene panel yielded 48 hits whose log2 fold change in mRNA expression met the selection criteria. Of these 48, 23 genes were found to be significant or show a trend of significance in hNSC‐EV treated AD mice (Figure 6A–D), and another 20 genes were differentially expressed by the iMGL‐EV treated AD group; the remaining 5 genes were significant in both EV‐treated cohorts. Box plot representation of NF‐κB, apoptosis, oligodendrocytes, and inflammatory signaling‐related genes showed downregulation of these genes in the AD brains treated with iMGL‐EVs compared to those treated with hNSC‐EVs brains (Figure S6).

In addition to individual gene expression analysis, we also quantified transcriptomic changes in 20 unique gene sets categorized by cellular functionality as defined by the analysis software provided with the RNA‐sequencing panel. The analysis used both weighted and non‐weighted gene expression levels to generate Pathway Scores and Directed Global Significance Scores, respectively, both of which were normalized to vehicle‐treated AD expression (see Figure S7A,B). Higher scores indicated upregulation of the gene set compared to AD vehicle mice, and lower scores signified downregulation. Compared to vehicle‐treated AD, astrocyte function, oligodendrocyte function, and apoptosis pathways were downregulated in iMGL‐EV‐treated AD mice but upregulated in AD mice receiving hNSC‐derived EVs. Concurrently, neurons and neurotransmission, insulin signaling, and carbohydrate metabolism pathways were upregulated in AD mice treated with iMGL‐EVs and downregulated in hNSC‐EV‐treated AD mice compared to vehicle AD.

### 3.6 hNSC‐ and iMGL‐Derived EVs Deliver Unique Biological Cargo to AD Brain

To compare the therapeutic cargo within hNSC‐ and iMGL‐derived EVs, samples from both EV types underwent RNA sequencing to generate separate miR profile libraries. From these libraries, miRs were selected whose total gene counts constituted more than 1% of total miR expression or were expressed in both library sets (Figure 6E–G). hNSC‐derived EVs yielded 28 unique miRs, while iMGL‐derived EVs yielded 15 uniquely expressed miRs. The topmost miR hits from hNSC‐derived EVs were less variable in individual expression levels, between 1.049% and 1.504% of total miR (Figure 6E), while miRs from iMGL‐derived EVs were more disproportionately represented, ranging from 1.107% to 19.984% of total miR expression (Figure 6F). Two miR species, miR‐1246 and miR‐3074, were expressed in EVs from both cell sources. Of these two, miR‐1246 was the more evenly represented in both hNSC‐ and iMGL‐derived EVs (1.113% and 1.107%, respectively). In contrast, miR‐3074 was minimally expressed in the hNSC‐derived EVs, at 0.009%. The functional enrichment analysis of miRs isolated from hNSC‐ (Figure 6e1) and iMGL‐derived (Figure 6f1) EVs is shown.

### 3.7 hNSC‐ and iMGL‐Derived EVs Reduce Proinflammatory Cytokine in 5xFAD Brain

To investigate the effects of EV treatment on inflammatory response chemokines in the Alzheimer's brain, a multiplex ELISA was performed to analyze cytokine levels in the brains of WT, AD + Vehicle, AD + hNSC‐EV, and AD + iMGL‐EV mice 10 weeks post‐EV treatment (Figure S8). Compared to WT, Interleukin‐6 (IL‐6) was significantly elevated in vehicle‐treated AD mice, and this increase was significantly reduced by hNSC‐EV treatment (*p* < 0.03). A similar trend was seen in AD mice treated with iMGL‐derived EVs. No significant differences were observed among the other cytokines across the experimental groups.

## 4 Discussion

### 4.1 Impact of hNSC‐ and iMGL‐Derived EVs on 5xFAD Brain

The key finding of this study is that EVs isolated from human iPSC‐derived microglia provide long‐term neuroprotective benefits in the AD brain, comparable to those of human neural stem cell‐derived EVs. This was reflected in improvements in object recognition memory, reductions in plaque numbers, microglial activation, gliosis surrounding plaques, and, importantly, the restoration of synaptic protein and neuroprotective gene expression in vivo. Previously, using a cytotoxic chemotherapy (doxorubicin)‐induced chemobrain model, we demonstrated a similar efficacy of iMGL‐derived EVs in alleviating cognitive deficits by attenuating microglial activation and reducing neuroinflammation (Allen et al. 2019). In this study, iMGL‐EVs exhibited functionally comparable neurocognitive benefits to the hNSC‐EVs, except for the distinct transcriptomic profiles observed in the 5xFAD brains. iMGL‐ and hNSC‐derived EVs also reduced proinflammatory cytokine IL‐6 levels in the AD brain. In our previous study, 5xFAD mice were injected with EVs at 2 or 6 months of age and evaluated for cognitive impairments and molecular pathology 1 month later. At that post‐EV treatment time, we observed some improvements in behavioral endpoints, as well as reduced numbers of Aβ plaques and inflammation measured by CD68, along with increased synaptophysin levels (Apodaca et al. 2021). Our current study significantly expands on these findings, demonstrating that four weekly EV treatments with either iMGL‐ or hNSC‐EVs in 3‐month‐old male 5xFAD mice slow disease progression and mitigate AD pathology for up to 4 months after the final EV treatment (corresponding to 9 months of animal age). We acknowledge that sex differences in human AD and mouse models have been reported. Female 5xFAD mice show significantly increased Aβ plaques in the hippocampus and entorhinal cortices compared to males, along with an elevated pro‐inflammatory microglial state characterized by a neurodegenerative phenotype (Lopez‐Lee et al. 2024; Rodriguez et al. 2025). These differences were not dependent on the estrus cycle. Conversely, PET imaging and CSF analysis revealed no sex differences in Aβ levels between males and females (Ferretti et al. 2018). To be consistent with our previous study (Apodaca et al. 2021), this research expanded the therapeutic approach using a multiple injection paradigm (once weekly for 4 weeks) and compared two different EV sources to determine their neuroprotective benefits in male 5xFAD mice exclusively. We also acknowledge that AD is a progressive, genotypic, and phenotypic disease with early, moderate, and severe stages of disease progression (Reitz and Mayeux 2014). Compared to other traditional models (3xTg, Arctic, etc.), the 5xFAD model, which incorporates five human familial AD gene mutations, exhibits accelerated disease progression. This includes neuronal beta‐amyloid aggregation within 6–8 weeks of age, which is then exacerbated by 8–12 weeks of age, resulting in amyloid and plaque accumulation. Overall, neurodegeneration and cognitive decline are evident at 6–8 months of age (Forner et al. 2021; Kimura and Ohno 2009; Oakley et al. 2006). As shown in our previous work (Apodaca et al. 2021) as well as in the work of others (Oakley et al. 2006), the 5xFAD model enables detection of disease progression at an early neurodegenerative stage and its long‐term effects on plaque accumulation and resulting neurodegenerative and neurocognitive sequelae. Therefore, we selected 12 weeks of age for our neuroprotective EV intervention to prevent long‐term neurodegeneration and cognitive impairments in the 5xFAD model.

### 4.2 Neuroinflammation and Synaptic Density

We evaluated plaque load in two cortical regions, the medial pre‐frontal cortex (mPFC) and the perirhinal cortex (PRh) of the AD brain following hNSC‐ and iMGL‐EV treatment. Both the mPFC and PRh play essential roles in recognition and recall memory, as well as the ability to discriminate between objects or spatial contexts (Barker et al. 2007; Barker and Warburton 2011). We found reduced plaque numbers in both regions in groups treated with iMGL‐ and hNSC‐EV, which persisted from 10 to 16 weeks post‐EV injections, corresponding to 9 months of animal age. This data indicated that EVs were able to prevent plaque deposition in an accelerated mouse model of AD (5xFAD). A similar strategy should be tested in the future using alternative AD models, including Arctic or 3xTg transgenic mice. Reduction of the presynaptic vesicle protein synaptophysin is one of the hallmarks of AD brain (Blurton‐Jones et al. 2009). Synaptophysin plays essential roles in the formation, trafficking, and endocytosis of synaptic vesicles, and its loss is linked with AD‐related neurodegeneration. Similarly, PSD‐95 is a crucial postsynaptic scaffolding protein that mediates the organization and functional regulation of excitatory neurotransmitter receptors within synaptic complexes (Glantz et al. 2007; Keith and El‐Husseini 2008). We observed restoration of synaptophysin and PSD‐95 immunoreactivity in the AD brain after treatment with either hNSC‐ or iMGL‐derived EVs, indicating a comparable neuroprotective effect in vivo. Furthermore, in the AD brain, elevated expression of CD9^+^ disease‐associated microglia (DAM) has been shown to contribute to neurodegeneration and neuroinflammation in the 5xFAD model and human postmortem brains (Keren‐Shaul et al. 2017). DAMs, located near plaques, are associated with pro‐inflammatory activation, elevated lipid metabolism, and phagocytosis in AD brains. We observed elevated CD9 and hypertrophic astrocytes surrounding plaques in the mPFC and PRh. This was accompanied by elevated phagocytic, activated microglia (CD68‐IBA1) in the AD brain. Prolonged proinflammatory activation of microglia can further damage the neuronal landscape, leading to synaptic loss. Additionally, we found elevated astrocytic expression of complement anaphylatoxin C3d in AD brains. Astrocyte hypertrophy and higher C3 expression are associated with exacerbated AD pathology, including Aβ clearance (Iram et al. 2016). hNSC‐ and iMGL‐EVs reduced DAMs, microglial activation, and astrocytic C3d expression in vivo, thereby preventing synaptic and cognitive decline in our study, indicating a neuroprotective effect of our regenerative approach. We recognize that future studies must assess systemic toxicity or effects on peripheral organs. However, we did not observe increased (neuro) inflammation, cytokines, or changes in synaptic proteins in the host animals.

### 4.3 Neuroinflammatory Gene Expression

To better understand the beneficial neuroprotective impact of EVs, we analyzed gene expression changes in the AD brain with or without EV treatments. NanoString neuroinflammation panel analysis revealed 48 gene signatures with a significant log2 fold change. Of the analyzed genes, four were upregulated in AD mice receiving either stem cell‐derived EV, including *Ago4*, *Gbp2*, *Tgm2*, and *Tnfsf10*. *Ago4* is regulated by *Mecp2*, a positive regulator of synaptic plasticity and cognitive function (Sarahian et al. 2025). *Mecp2* (Methyl‐CpG‐binding protein 2), in turn, regulates several neuronal function pathways, including transcription of ligands, receptors, ion channels, and DNA methylation. Our pathway analysis (Figure 6f1) showed that iMGL‐EVs were enriched with miRNAs that influence TGFβ signaling, reparative microglial responses, cell proliferation, cell cycle, chemotaxis, angiogenesis, and the negative regulation of NF‐kB signal transduction. *Gbp2* (Guanylate‐binding protein 2) is linked to microglial function and associated with the NF‐κB pathway (You et al. 2024). Thus, tissue *Gbp2* expression could be related to an adaptive response against the EV‐derived miRNA target (i.e., the NF‐kB signal transduction pathway). Likewise, the 5xFAD model is marked by the rapid deposition of Aβ plaques that serve as “seeds” to accelerate tau aggregation. *Tgm2*, tissue transglutaminase, is implicated in AD, and it has been shown to cross‐link amyloid‐beta and tau proteins, leading to further aggregation (Min and Chung 2018). Following EV‐mediated reductions in Aβ plaque, as we observe here, elevated transglutaminase (*Tgm2*) expression or activity could be postulated as a compensatory mechanism to capture the remaining substrate in the microenvironment. These possibilities still need to be confirmed experimentally. Additionally, hNSC‐EV treatment elevated *Gzma* and reduced *Gzmb* (granzyme) expression. GzmA has been shown to cleave tau proteolytically, and GzmB is linked with senescence and cytotoxicity in the AD pathogenesis (Dressman et al. 2022; Quinn et al. 2024). iMGL‐EV treatment reduced *Bcas1* expression, which plays essential roles in early oligodendrogenesis and myelination (Fard et al. 2017). Notably, iMGL‐EVs significantly downregulated other inflammatory signaling genes, including *Nfkbie, Ptger4*, and *Serping1*. *Nfkbie (*NF‐kB light polypeptide gene enhancer), a member of the NF‐κB suprafamily, is associated with neurotoxicity following astrogliosis (Jong Huat et al. 2024). *Serping1* (serpin family G member 1) regulates the enzymatic activation of the classical complement cascade pathway and is found to be elevated in AD brains (Heit et al. 2013; Zattoni et al. 2022). *Tnfsf10* (TNF suprafamily member 10), a proinflammatory cytokine‐related gene of the TNF superfamily, has been shown to modulate immune response by infiltrating macrophages and microglia in the AD brain (Cantone et al. 2025). *Tnfsf10* expression was increased following hNSC‐ or iMGL‐EV treatments. On the other hand, treatment with iMGL‐EV downregulated the expression of *Map3k14* (mitogen‐activated protein kinase), a pro‐inflammatory gene relevant to AD pathogenesis and the TNF pathway (Rogers et al. 2024; Rudnitskaya et al. 2022). We acknowledge that we did not validate these mRNA by qPCR or conduct protein analysis. Thus, the confirmation of a protein or peptide product will provide more information on specific pathways and gene functions in vivo. Gene set analysis indicated that in comparison to the vehicle‐treated AD brain, astrocyte function, oligodendrocyte function, and apoptosis pathways were downregulated in iMGL‐EV‐treated AD mice. On the contrary, these genes were upregulated in AD brains treated with hNSC‐EV. iMGL‐EV also upregulated neuron function, insulin signaling, and carbohydrate metabolism pathway‐related genes, whereas hNSC‐EVs downregulated these genes in the AD brain. We also found an increase in hypertrophic astrocytes and DAMs surrounding plaques, and microglial activation in the vehicle‐treated AD brain, which was significantly reduced following iMGL‐EV treatment. Overall, these results demonstrate the neuroprotective and anti‐inflammatory effects of iMGL‐EV treatment in AD brains.

### 4.4 EV‐Derived miRNA Cargo

In addition to proteins, lipids, and mitochondrial components, miRs are often found in EV cargo. While distinct neural stem cells and differentiated microglia secrete a variety of EVs containing unique cargo, the most common proteome profiles include tetraspanins, ribosomal, mitochondrial, and cytoskeleton component proteins, metalloproteases, and SNARE components (soluble NSF attachment protein receptor) (Jeppesen et al. 2023; Lischnig et al. 2022; You et al. 2022). In our study, we injected EVs once weekly for 4 weeks and analyzed their impact on neurobiological and neuropathological parameters at 10‐ and 16‐week post‐treatment. We postulated that EV and EV cargo‐derived proteins would have been metabolized during this prolonged period, and that miRs would have exerted functional effects on multiple pathways in vivo. Concurrently, we observed significantly reduced levels of IL6, DAMs, and gliosis in AD brains following treatment with hNSC‐ and iMGL‐EVs, indicating an anti‐inflammatory effect of EVs and EV‐derived cargo, including miRs. Thus, we performed miR sequencing of hNSC‐ and iMGL‐EV‐derived cargo. Both types of EVs exhibited unique miR signatures. hNSC‐EVs cargo contained 30 primary expressed miRs (each making up ≥ 1% of total miR expression). hNSC‐derived EVs had approximately equal distribution (~1.5%) of 26 miRs, with the highest expression of miR‐3940 species (1.504%). Conversely, iMGL‐EVs had six majorly expressed miRs, including miR‐16 (19.984%), miR‐23a (11.254%), miR‐191 (7.799%), miR‐146a (6.029%), miR‐26a (4.705%), and miR‐122 (4.561%). Two miR species, miR‐1246 and miR‐3074, were expressed in EVs from both cell sources. miR‐1246 plays a significant role in cell proliferation, differentiation, and apoptosis (Ghafouri‐Fard et al. 2021). miR‐1246 influences downstream targets such as TP53, GSK (glycogen synthase kinase), transcription factor YY1, and NF‐κB. Notably, EV‐derived miR‐1246 promotes differentiation and reduces activation and function of myeloid‐derived suppressor cells (MDSCs) (Qiu et al. 2021). miR‐1246 also plays a regulatory role in *Tnfsf10* expression in the canonical Wnt signaling (Worst et al. 2017), and the disruption of canonical *Wnt* signaling exacerbates AD pathogenesis (Tapia‐Rojas and Inestrosa 2018). miR‐3074 has been shown to play a role in myoblast differentiation via interacting with Cav1 (Caveolin 1) miRNA (Lee et al. 2020). Cav1 promotes myogenesis. Additionally, miR‐3074 is also expressed in the human uterine decidual tissue and plays a role in fetal development (Meng et al. 2021). miR‐3074‐5p activated NLRP3 (NOD‐like receptor family, pyrin domain‐containing 3) inflammasome in macrophages and led to a feedback elevation of anti‐inflammatory effectors (Yang et al. 2024). We did not directly validate the downstream targets for miRNAs‐1246 and ‐3074. One common pathway targeted by both miRs is inflammation. Our analysis of neuro‐inflammatory markers, including DAMs (Figure 4), activated microglia (Figure 5), and the neuroinflammation‐related gene expression (Figure 6), showed reduced inflammation in the EV‐treated AD brains. Together, these results suggest a neuroprotective effect of EV or EV‐derived miR cargo in reducing inflammation in vivo. Given the comparatively low abundance of miR‐3074 in hNSC‐ versus iMGL‐derived EVs (0.009% and 1.266%, respectively), we propose miR‐1246 as the more promising effector candidate, which showed a more even abundance in both EV sources. In addition to these two common miRs, each cell line expresses unique miRs with neuro‐modulatory functions in vivo. iMGL‐EV derived cargo contained miR‐16, miR‐23a, and miR‐191. miRNA‐16 plays important roles in brain development and synaptic function (Burak et al. 2018). Importantly, miR‐16 has been shown to reduce BACE1 (beta‐site amyloid precursor protein cleaving enzyme 1, or β‐secretase 1) expression and facilitate amyloid beta‐induced neurotoxicity and apoptosis in vitro (Burak et al. 2018). miR‐23a has been shown to suppress lamin B1 and increased oligodendrocyte differentiation and promote healthy myelination (Lin et al. 2013). miR‐23a was protective in TBI (traumatic brain injury) and focal cerebral ischemia models by reducing apoptosis, inflammation, and oxidative stress (Li, Xu, et al. 2020; Zhao et al. 2014). miR‐23a upregulation has also been linked to GranB (*Gzmb*) suppression and reduction in cytotoxicity (Lin et al. 2014). miR‐191 regulates BDNF and promotes spine remodeling and plasticity (Hu et al. 2014). Like miR‐23a, miR‐146a is correlated with and has high binding affinity for GranB, as well as for GranA (*Gzma*) (Chen et al. 2023; Kim et al. 2024). Thus, unique yet neuroprotective miR targets and downstream pathway could contribute to the EV‐mediated beneficial functional impact in the AD brain.

### 4.5 Functional Enrichment Analysis

Functional enrichment analysis of miRs isolated from iMGL‐EV revealed a strong association with signaling networks that regulate cellular activation and immune modulation (Figure 6e1,f1). The top enriched pathways included regulation of the ERK1/ERK2 (extracellular signal‐regulated kinases) and MAPK (mitogen‐activated protein kinase) cascades, TGFβ signaling, cytokine‐mediated signaling, SMAD (Sma and Mad‐related) protein signal transduction, and canonical NF‐κB activation. These pathways collectively reflect the functional targets of iMGL‐EV‐derived miRs to modulate key transcriptional and inflammatory circuits in the host cells. In addition, “response to growth factor” and “miRNA involved in DNA damage response” suggest that these EV‐borne miRs contribute to cellular stress adaptation and repair mechanisms. Together, these results indicate that iMGL‐derived EVs harbor miRs with the potential to orchestrate signaling cascades linked to inflammation, stress, and cell survival, highlighting their broader immunoregulatory role in the neural microenvironment.

In contrast, miRs identified from hNSC‐EVs showed predominant enrichment in pathways related to ncRNA‐mediated, post‐transcriptional, and miR‐mediated gene silencing. This suggests that hNSC‐derived EV miRNAs mainly function as regulators of mRNA stability and translation, contributing to transcriptomic homeostasis rather than direct activation of signaling pathways. The shared enrichment of RNA‐silencing processes across both EV types underscores a conserved post‐transcriptional regulatory mechanism mediated by EV‐associated miRs. However, the stronger enrichment of cytokine, TGFβ, and MAPK signaling pathways in the iMGL‐derived EVs suggests a more pronounced immunomodulatory and stress‐responsive function, whereas hNSC‐EVs appear to maintain a predominantly gene‐regulatory and neuroprotective profile. Collectively, these findings suggest complementary yet distinct biological functions of EV‐derived miRs in modulating intercellular communication within the neural microenvironment. Overall, we hypothesize that the beneficial neuro‐modulatory miR cargo facilitated cognitive recovery and reduced neurodegenerative events in our AD model. Nonetheless, the functional impact of individual miR cargo and their downstream targets needs to be confirmed to determine the molecular mechanism(s) behind the benefits of EV treatment.

## 5 Conclusion

Our study demonstrates the beneficial neurocognitive impact of a cell‐free, regenerative approach using hNSC‐ and iMGL‐derived EVs. We found functional equivalence of iMGL‐EV comparable to that of hNSC‐EV, with noted differences in the gene expression and miR cargo content. Our findings also demonstrated the neuroprotective efficacy of repeated systemic EV injections in reducing AD‐related cognitive impairment and neurodegenerative sequelae. Additionally, our data indicate unique miR signatures in the EV‐derived cargo, suggesting specific pathways and mechanisms that need further validation to understand their functional impact on the AD brain to facilitate the translational application of this approach.

## Author Contributions

Munjal M. Acharya and Janet E. Baulch conceptualized and designed the study. Robert P. Krattli Jr., Mineh Markarian, Shreya Madan, and Amanda McQuade developed methodology. Robert P. Krattli Jr., Mineh Markarian, and Shreya Madan acquired data. Robert P. Krattli Jr., Shreya Madan, Devyani Swami, Janet E. Baulch, and Munjal M. Acharya analyzed and interpreted the data. Robert P. Krattli Jr., Shreya Madan, Amanda McQuade, Janet E. Baulch, and Munjal M. Acharya wrote, reviewed, and/or revised the manuscript. Amanda McQuade, Matthew Blurton‐Jones, Janet E. Baulch, and Munjal M. Acharya provided administrative, technical, or material support. Munjal M. Acharya, Matthew Blurton‐Jones, and Janet E. Baulch supervised the study.

## Funding

This work was supported by California Institute for Regenerative Medicine, DISC1‐10079, DISC2‐12400. UC Irvine Sue and Bill Gross Stem Cell Research Center. U.S. National Institutes of Health, R01CA262213.

## Conflicts of Interest

The authors declare no conflicts of interest.

## Supporting information


**Appendix S1:** acel70341‐sup‐0001‐AppendixS1.docx.

## Data Availability

The datasets generated during and/or analyzed during the current study are available from the corresponding author upon request. The Gene Expression Omnibus (GEO) reference number for the presented RNA sequencing study is GSE285754.
