# Supplementary material for: Comparing Functional Consequences of Human iPSC‐Microglia and Neural Stem Cell‐Derived Extracellular Vesicles in Mitigating Cognitive Decline in Alzheimer's Disease
Source: Aging Cell. 2025 Dec 23;25(1):e70341. doi: 10.1111/acel.70341 (PMC12723720; doi:10.1111/acel.70341)
Supplement: Supplementary file 1 — Appendix S1: acel70341‐sup‐0001‐AppendixS1.docx. [file ACEL-25-e70341-s001.docx]

**SUPPLEMENTAL INFORMATION**

**Supplemental Figure S1:**

**
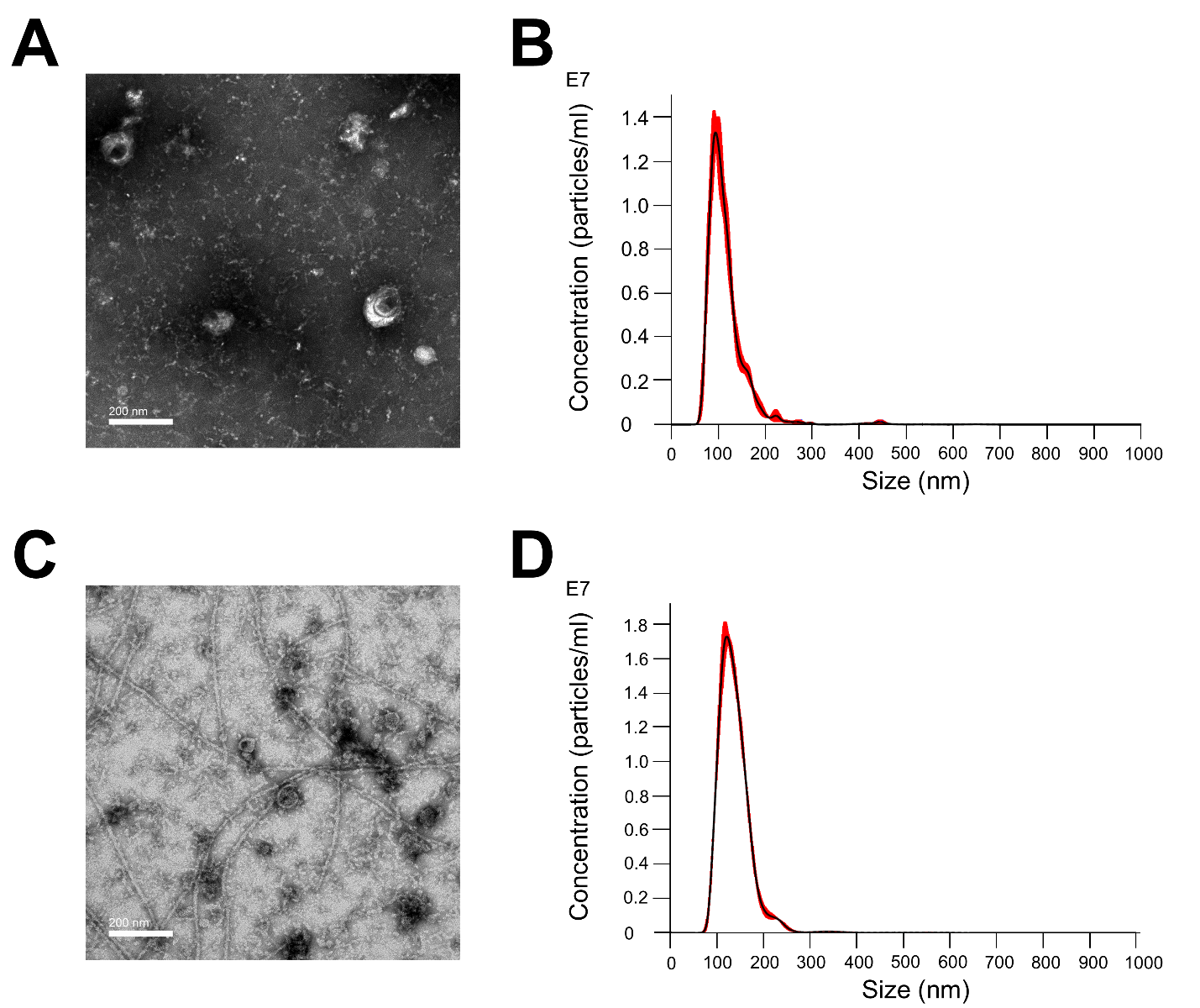
**

**Supplemental Figure S1:** **Characterization of hNSC- (A-B) and iMGL- (C-D) derived extracellular vesicles.** (**A and C**) Transmission electron micrographs of general morphology and relative size of EVs (courtesy of UCSD-CMM-EM Core, San Diego). (**B and D**) A graphical representation of EV size distribution and concentration acquired from nanoparticle tracking analysis (NanoSight, Linear Spectroscopy Lab, UC Irvine). Scale bar, 200 µm (**A and C**).

**Supplement Figure S2:**

**
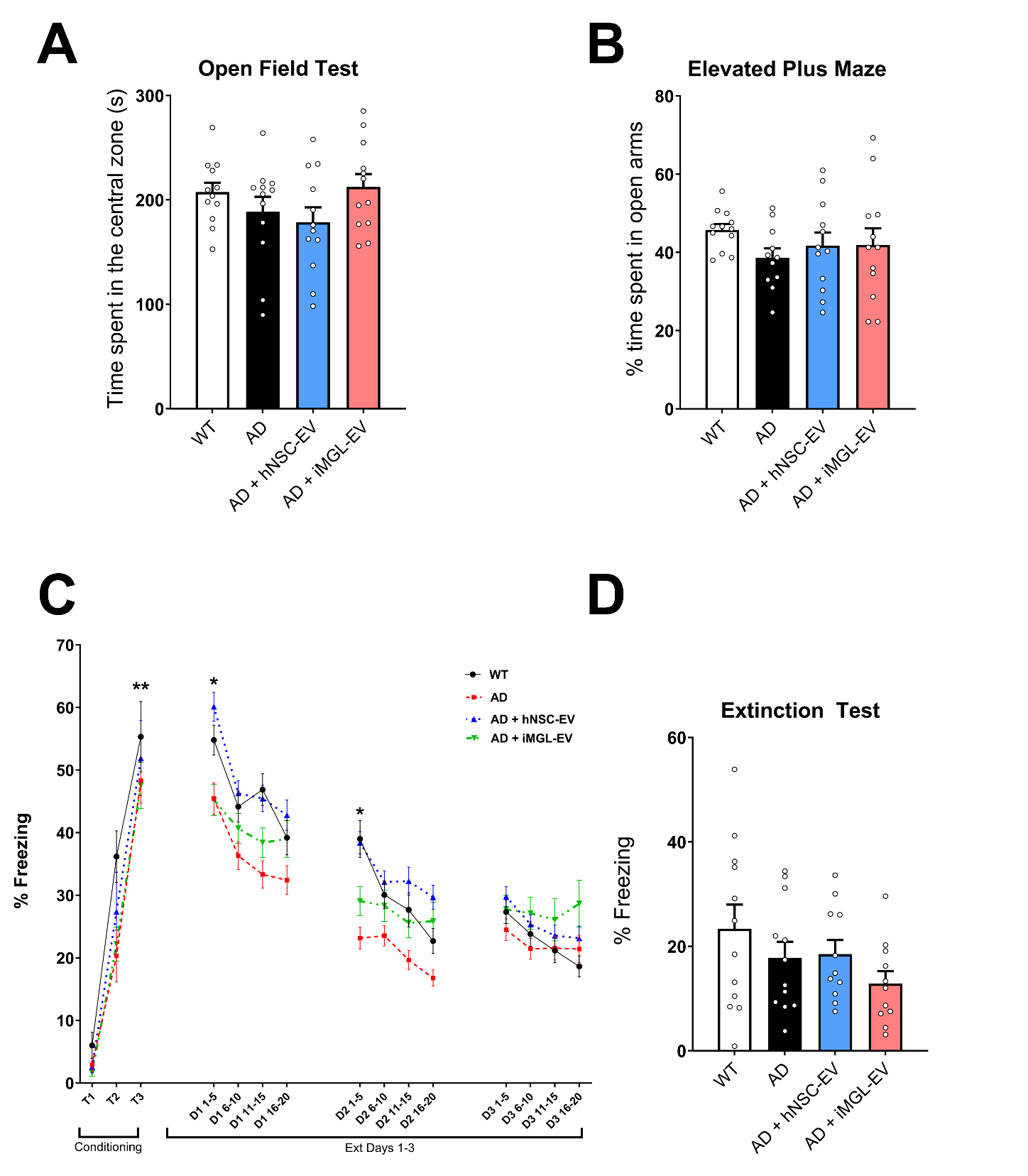
**

**Supplemental Figure S2:** **Behavior of 5xFAD mice and hNSC- and iMGL-derived EV treatment on anxiety (A-B) and fear memory consolidation (C-D) tasks.**

(**A**) The Open Field Test (OFT) test was performed on the first day of habituation for the Object Recognition Memory (ORM) test. The central zone was defined as the inner 60% square area of the total arena box; the percentage of time explored did not differ significantly across groups. (**B**) In the elevated plus maze task (EPM), the time spent in the open arms was not statistically significant between the experimental groups, indicating an absence of anxiety-like behavior. (**C**) For the fear extinction task, neither vehicle-treated AD nor EV-treated AD groups interfered with the acquisition of the fear conditioned response, as shown by the increase in freezing levels following a series of three-tone and shock pairings (80 dB, 0.6 mA, T1–T3). The data points for the extinction training phase (Days 1-3) are presented as binned averages of percent time freezing per 5 tone intervals (20 tones total per day). All experimental groups showed a gradual decrease in time spent freezing (Ext Days 1-3). However, on day 1 and 2 of extinction training, both WT and iMGL-EV treated AD groups showed an increase in time spent freezing as compared to vehicle treated AD. (**D**) 24h after day 3 of extinction training, the animals underwent the extinction test. WT, AD+iMGL-EV, and AD+hNSC-EV groups showed no statistical significance in the freezing levels compared to the AD group. Data is presented as mean ± SEM (N=12-16 mice per group). *P* values were derived from one-way ANOVA and Bonferroni’s multiple comparisons test. **P* < 0.01, versus AD. ** *P* < 0.0001, T1 versus T3 for all experimental groups.

**
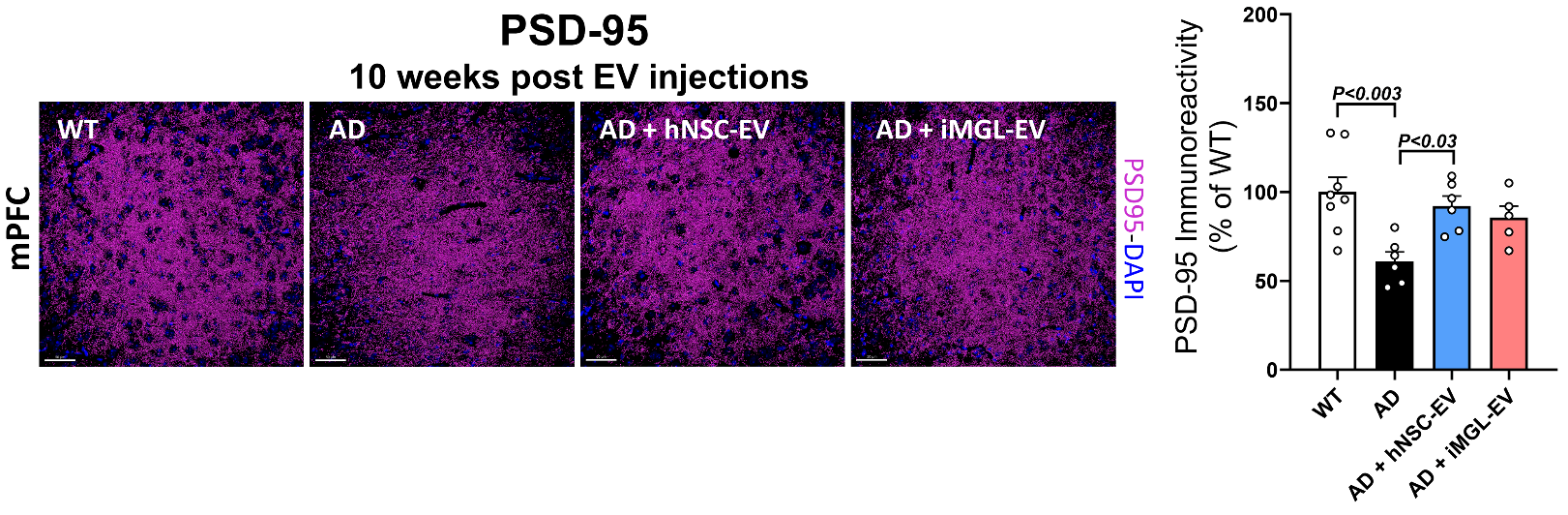
Supplemental Figure S3:**

**Supplemental Figure S3. hNSC-derived EVs Prevent PSD-95 Loss in Alzheimer’s Disease Mice mPFC.**

Representative images and quantification of the post-synaptic density protein, PSD-95, immunoreactivity (magenta; blue, DAPI nuclear counterstain) for the early time interval (at 10 weeks) after the last EV treatment. Immunofluorescence staining, laser scanning confocal microscopy, and volumetric image analysis were performed in the medial prefrontal cortex (mPFC). A significant decrease in PSD-95 immunoreactivity was found in the vehicle-treated AD mPFC compared to the wildtype (WT) controls. In contrast, hNSC-EV treatment increased PSD-95 compared to the vehicle-treated AD mice. iMGL-EV treatment showed a trend of increase in PSD-95 that was not statistically significant compared to the vehicle-treated AD group. Data is presented as Mean ± SEM (N = 3-4 mice/group), ANOVA, and Bonferroni’s multiple comparison test. Scale bar, 50 μm.

**
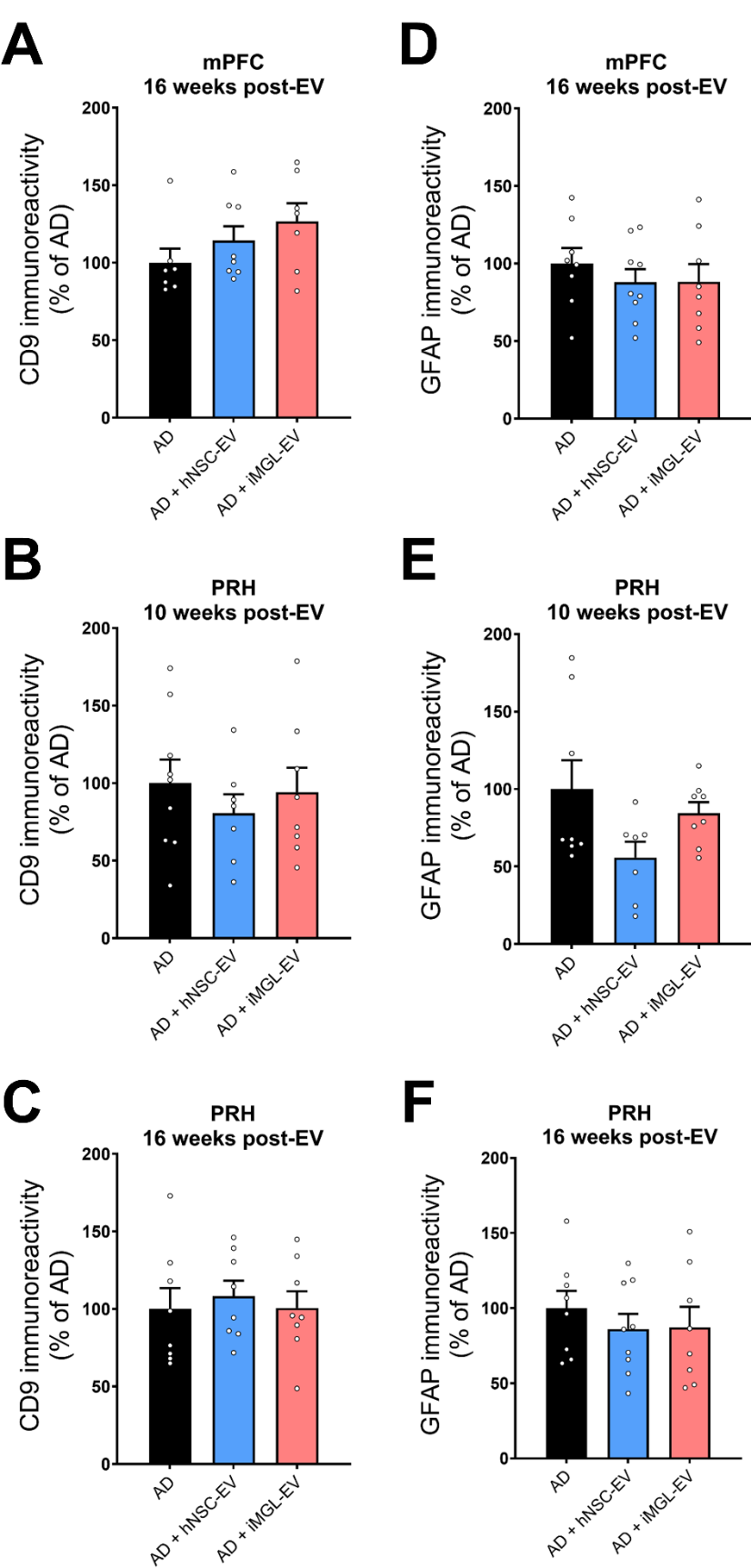
Supplemental Figure S4:**

**Supplemental Figure S4: Volumetric quantification of CD9 (A-C) and GFAP (D-F) immunoreactivity in the three AD treatment groups.** (**A** and **D**). There was no significant difference in expression for either marker in the medial prefrontal cortex (mPFC) from the late time interval (16-weeks post EV treatment). There was also no statistical significance for either marker in the perirhinal cortex (PRh) region at either 10 weeks (**B** and **E**) or 16 weeks (**C** and **F**) post-treatment. Data is presented as Mean ± SEM (N = 4 mice/group), ANOVA, and Bonferroni’s multiple comparison test.

**
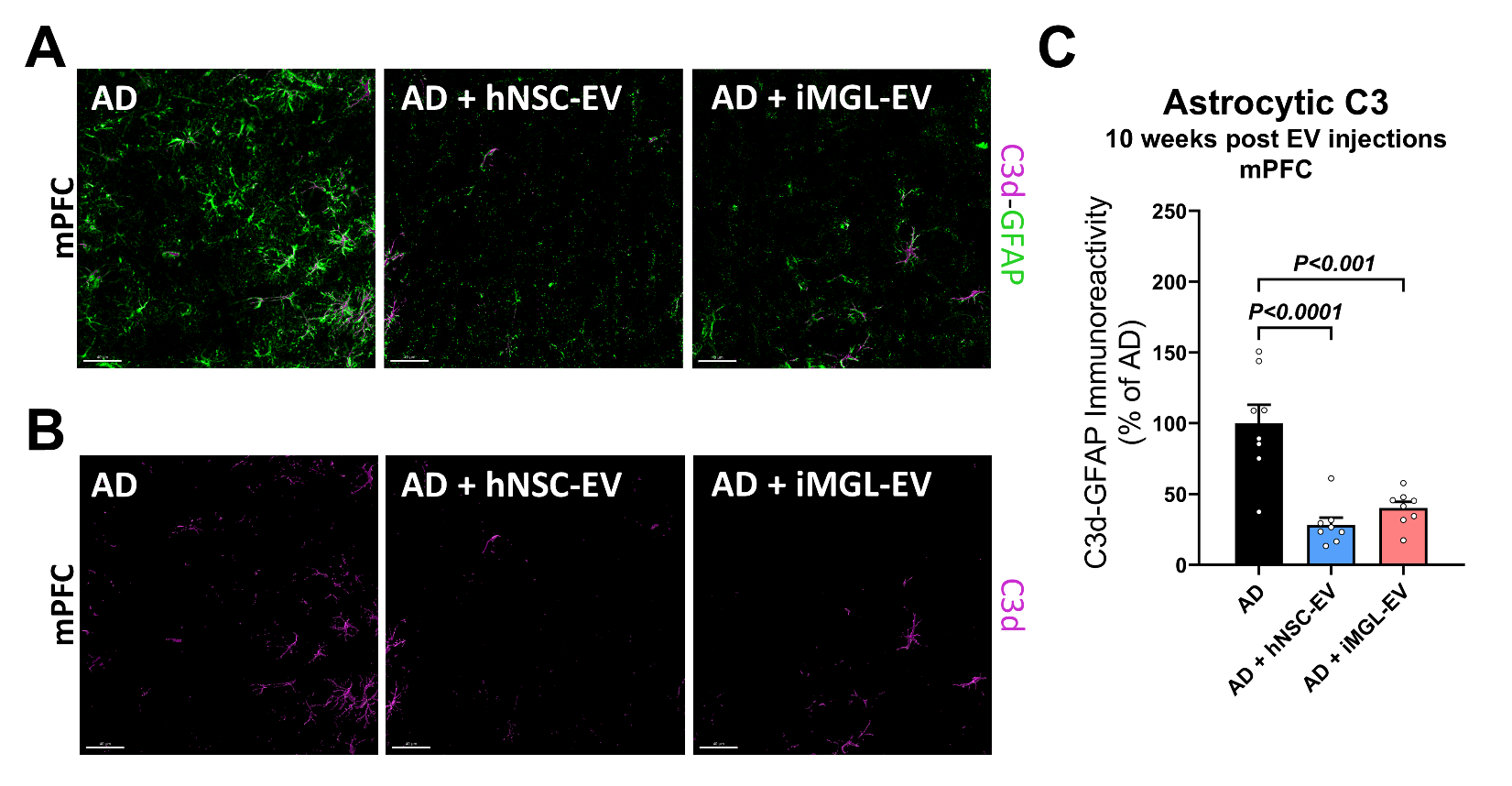
Supplemental Figure S5:**

**Supplemental Figure S5: EVs reduces astrocytic activation in the AD brain.**

**(A)** Representative images and volumetric analysis of astrocytic (GFAP^+^, green) colocalization of complement component C3d (magenta) in the mPFC regions of experimental groups at 10 weeks post EV treatment. **(B)** A separate magenta channel for C3d is shown for each group. Astrocytic C3d expression was quantified via the colocalized immunofluorescent expression C3d (magenta) with GFAP (green). **(C)** AD mice treated with both hNSC- and iMGL-derived EVs showed a significant decrease in C3d-GFAP colocalization compared to vehicle treated AD mice. Data is presented as Mean ± SEM (N = 4 mice/group), ANOVA, and Bonferroni’s multiple comparison test. Scale bars, 40 μm **(A-B)**.

**Supplemental Figure S6:**


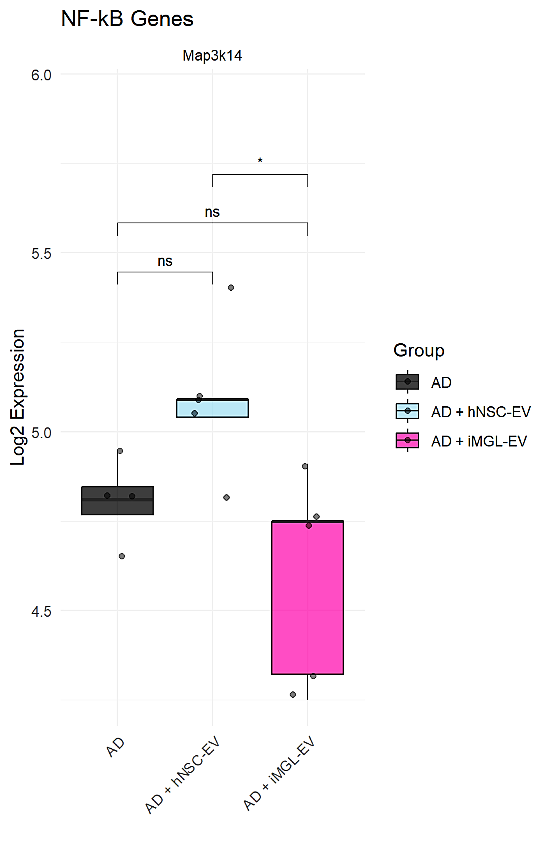


**A**


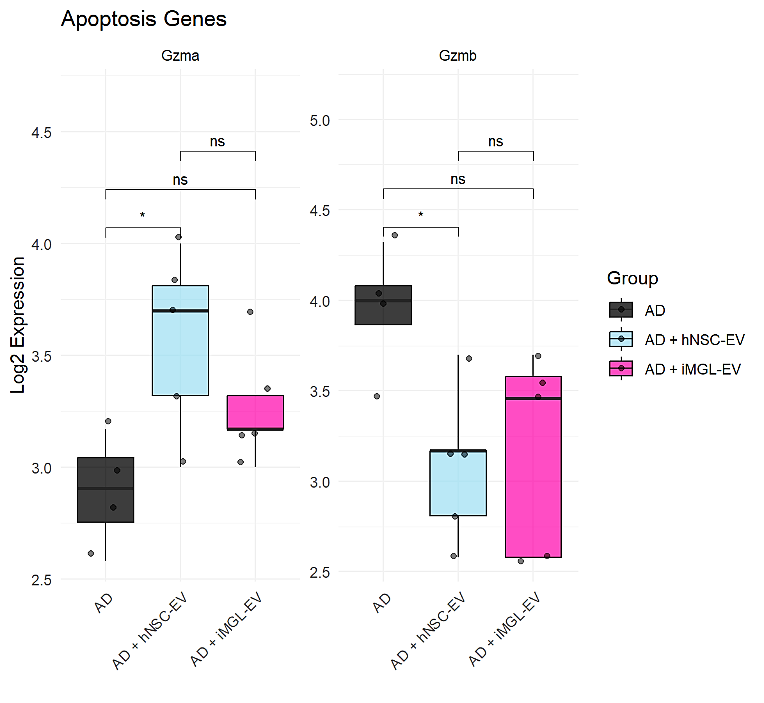


**B**


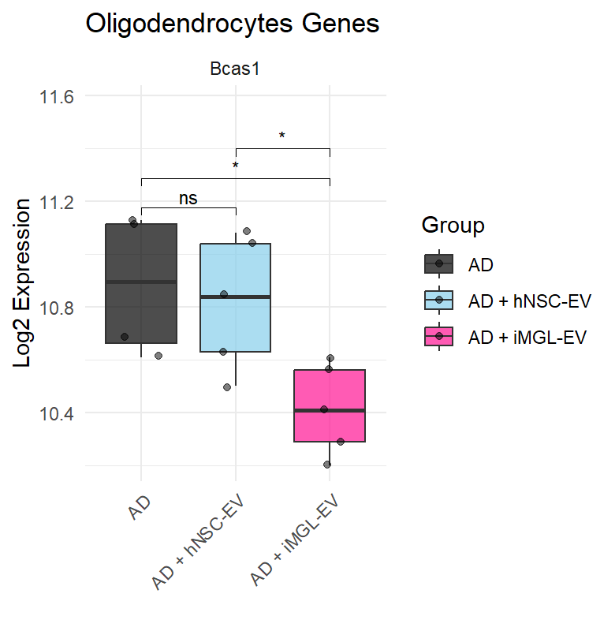

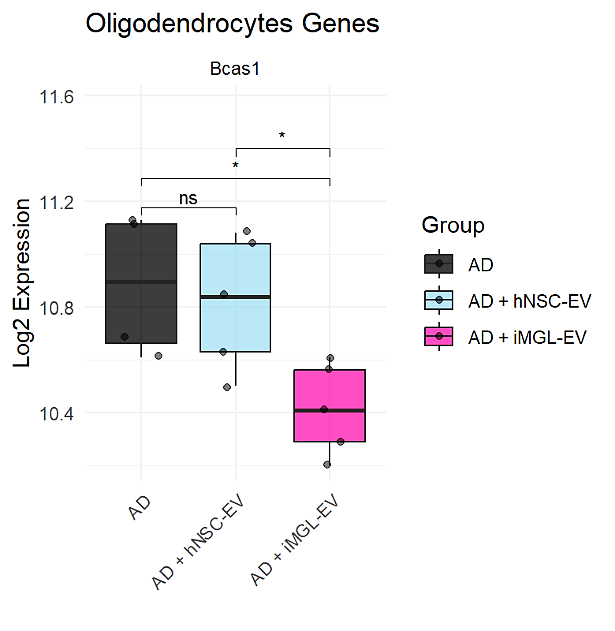


**C**


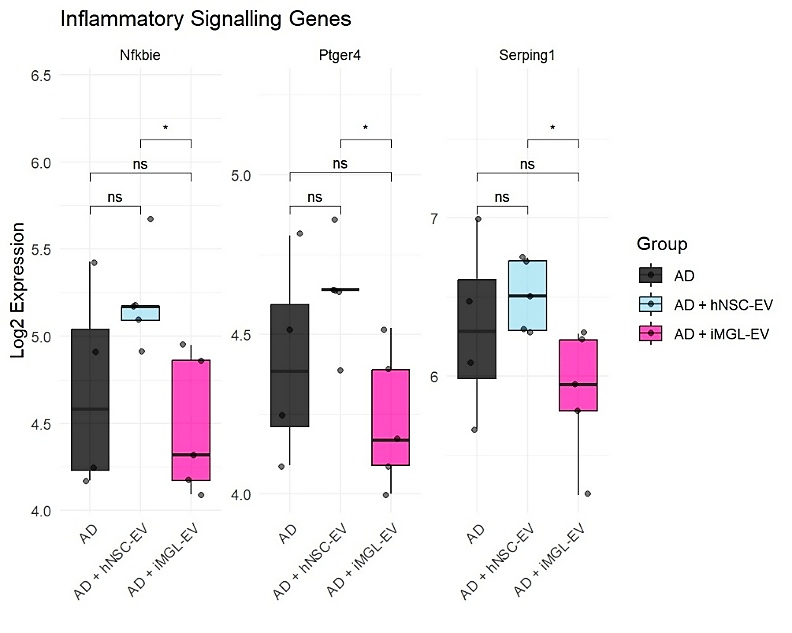


**D**

**Supplemental Figure S6:** **Boxplots showing the Log2 expression of specific genes** from **(A)** NF-ƙB signaling pathway, **(B)** Apoptosis pathway, **(C)** Oligodendrocyte genes and **(D)** Inflammatory Signaling markers. Data is presented as Mean ± SD (N = 4-5 mice/group), Mann–Whitney U test comparing the median of the experimental group. **P* < 0.05

**
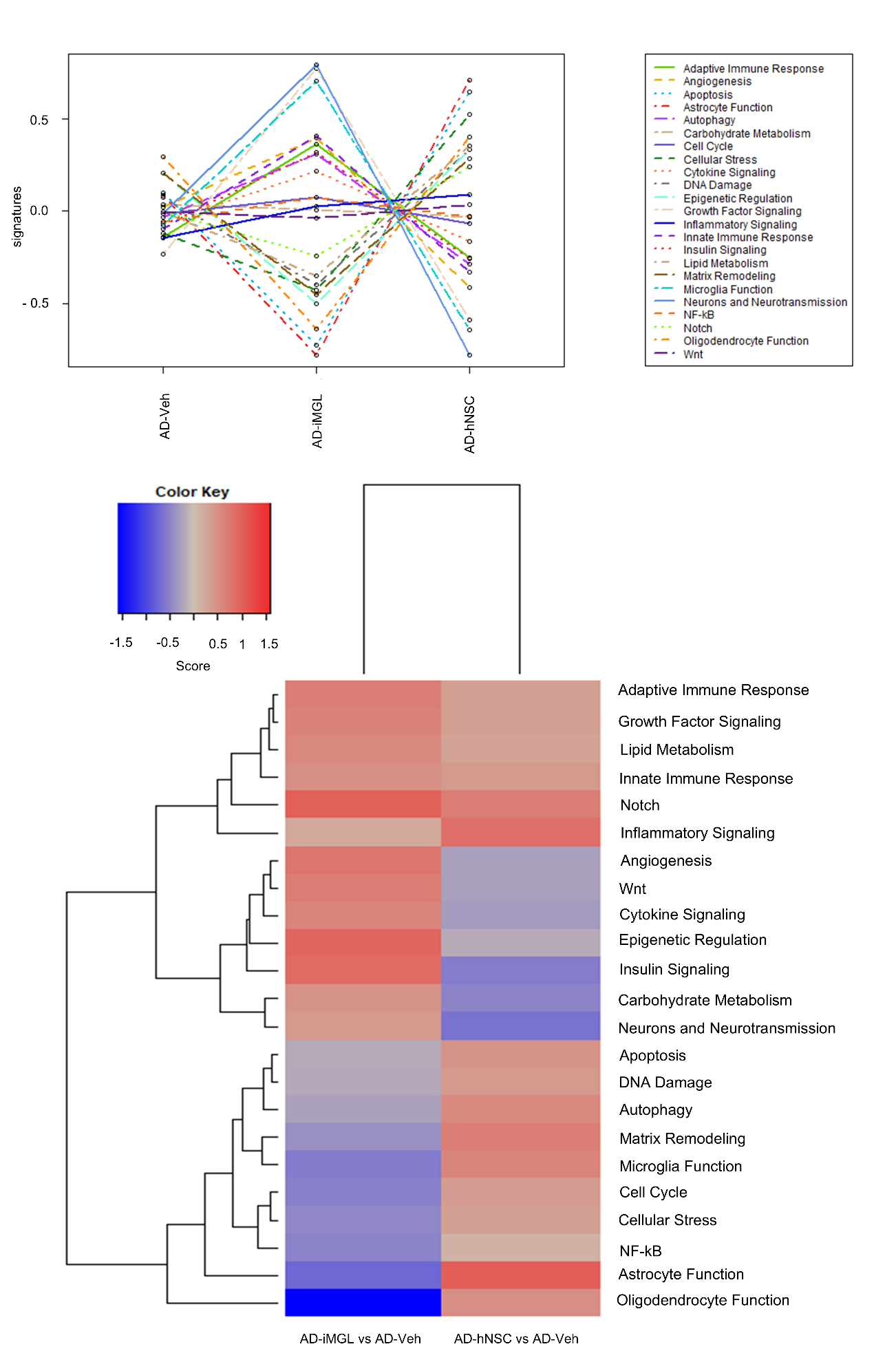
Supplemental Figure S7:**

**A**

**B**

**Supplemental Figure S7:** **Neuroinflammation transcriptomic profile and gene set pathways in 5xFAD mice treated with EVs.** 757 genes from a Neuroinflammation Panel (NanoString^TM^) were organized into 20 distinct pathways. Expression values are normalized to vehicle-treated AD mice. (**A**) Line plot of pathway signature scores showing the variation in expression of gene sets across experimental groups. (**B**) The heatmap displays the directed global significance scores reflecting the effect of hNSC- and iMGL-EVs on gene regulation in AD compared to vehicle-treated AD; red denotes over-expression; blue denotes reduced expression. Data is presented as Mean ± SEM (N = 4 mice/group)

**Supplemental Figure S8:
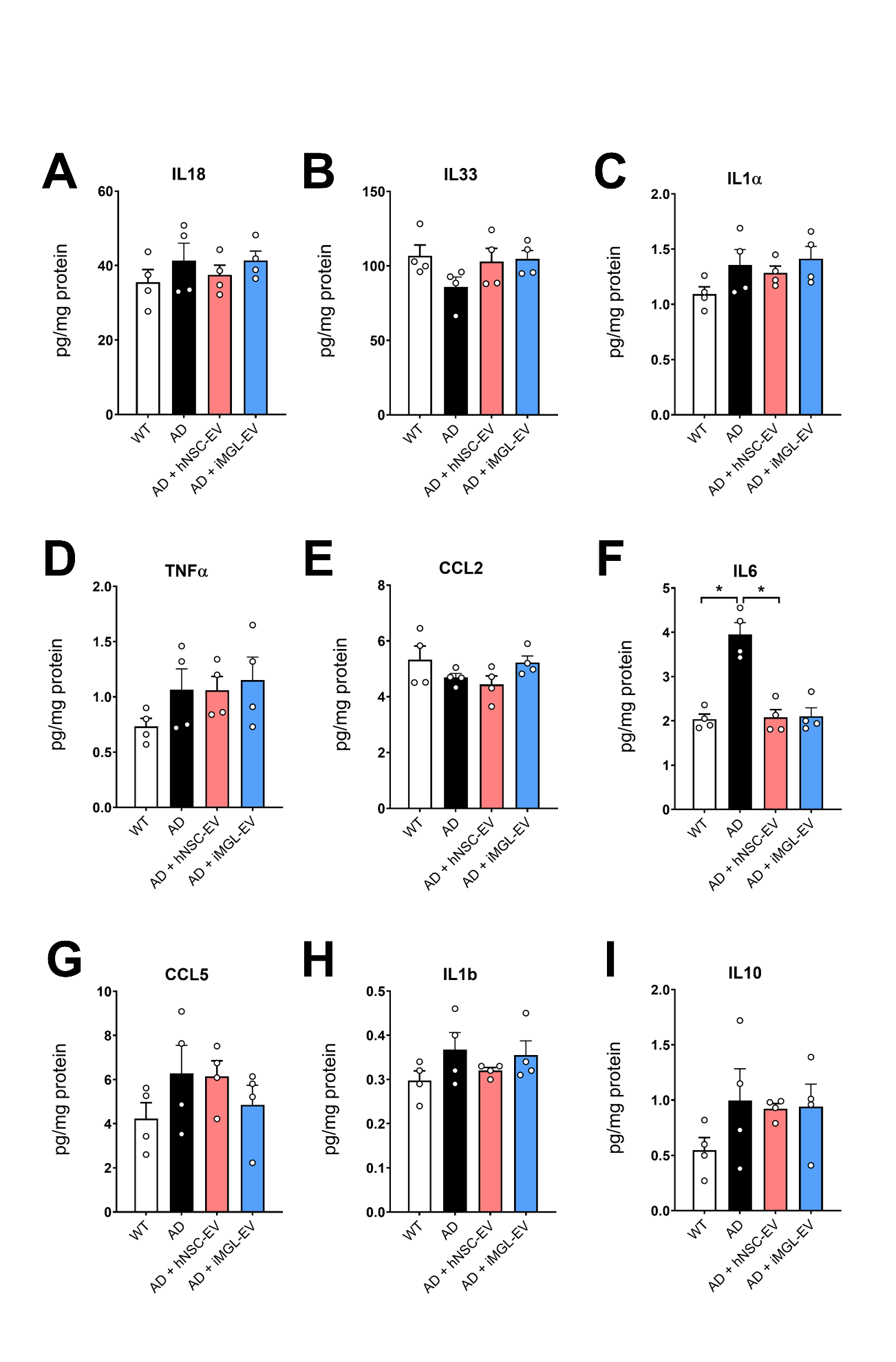
**

**Supplemental Figure S8: Multiplex ELISA analysis of cytokine levels** in brains of WT, AD, AD+hNSC-EV, and AD+iMGL-EV experimental groups. Brain samples extracted at the early time interval (10 weeks post-EV treatment). There was no observed statistical significance (*P* < 0.05) except for IL-6 **(F)**, which demonstrated elevated levels in the vehicle-treated AD group compared to WT and hNSC-EV-treated AD. A similar trend was observed for the AD+iMGL-EV group. Data is presented as Mean ± SEM (N = 4 mice/group), Kruskal-Wallis test, and Dunn’s multiple comparisons test.

**SUPPLEMENTAL METHODS AND MATERIALS**

**Generation, Isolation, and Characterization of hNSC- and iMGL-derived EVs**

The validation, expansion, and characterization of proliferating human neural stem cells (hNSCs, ENStem-A line, EMD Millipore) followed prior published procedures (Apodaca et al., 2021). Conditioned media collected from cultured iMGL cells was provided by Dr. Mathew Blurton-Jones (University of California, Irvine). Briefly, human iPSC-derived microglia were differentiated by simplified methods from human iPSC-derived mesodermal hematopoietic stem cells as described (McQuade et al., 2018). Blurton-Jones lab has conducted detailed RNA sequencing and characterization to functionally validate microglial characteristics during the differentiation phase (days 28 to 35). Before the extracellular vesicles (EVs) were isolated from each cell line, each conditioned culture medium was centrifuged first for 5 minutes at 300g and then again (with the supernatant from the first spin) for 20 minutes at 2500g. Then, the supernatant was collected and purified for each conditioned culture medium by filtering through a 0.22-μm sterile membrane. EVs were then isolated and further purified from each culture medium by ultracentrifugation. Each conditioned media was first transferred to a 70mL ultracentrifuge tube and centrifuged at 105,000g for 90 minutes. The pellet was then collected and washed with phosphate-buffered saline (PBS), then resuspended with PBS, and was then pooled into one 26 mL centrifuge tube. This was then spun at 105,000g for 2 hours. Finally, the pellet was resuspended with PBS/hybridization buffer before being aliquoted (400-500μl/cryovial). After isolating EVs, their size and concentration were characterized using nanoparticle tracking analysis (NTA) on a NanoSight NS300 particle analyzer (Laser Spectroscopy Laboratories, UCI). Approximately 600 µL of the EV stock diluted in PBS -/- was loaded into the sample carrier cell and was cleaned with PBS -/- between different EV cell sources. Data collection involved three 60-second recordings per sample, from which mean particle size and EV concentration (particles/mL) were determined by averaging all three recordings.

EVs were also analyzed by transmission electron microscopy (TEM, JEOL 1400 plus, bottom mounted with the Gatan one-view camera) for morphology at the Cellular and Molecular Medicine Electron Microscopy Core, University of California, San Diego (UCSD-CMM-EM Core, RRID: SCR_022039). The specimen was prepared for negative staining for TEM (Timo negative staining) by first covering a glass plate with parafilm. A 400-mesh copper grid coated with formvar and copper films was used to mount the tissues. 5μl of the specimen was placed onto the parafilm slide and then covered with a freshly glow-discharged grid (carbon face down). The specimen was then left to float for 10 minutes. The grid was then washed 3 times with 200μl of double distilled water (DDW) and then transferred to 2% uranyl Acetate in DDW for 1 minute. Excess staining was removed by using Whatman filter paper 1 to blot the grid for 3 seconds. Finally, the grid was air-dried using tweezers before imaging. For imaging, one electron microscopy (EM) grid was prepared for each sample in which 5-10 areas were selected that appeared to have adequate staining levels. Images were taken at 4kX, 10kX, and 40kX magnifications.

**Mice**

All animals used in this study were cared for in accordance with the guidelines provided by NIH and approved by the Institutional Animal Care and Use Committee (IACUC) at the University of California, Irvine. Male 5xFAD mice aged 12 weeks (RRID:MMRRC_034848-JAX) and age-matched littermate controls (RRID:IMSR_JAX:000664) were obtained from The Jackson Laboratory and housed in standard conditions (20 °C±1 °C; 70% ± 10% humidity; 12h:12h light and dark cycle) in groups of 2-4 mice per cage. The 5xFAD mouse model was utilized for its early onset of AD neuropathology, specifically the deposition of beta-amyloid plaques, neuronal loss, and cognitive impairments.

**EV Delivery**

Male 5XFAD mice and their wildtype littermate controls were divided into the following groups: vehicle-injected AD with hibernation buffer (Gibco; AD; N=12), iMGL-EV-injected AD (AD+iMGL-EV; N=14), hNSCs-EV-injected AD (AD+hNSC-EV; N=14), and vehicle-injected wild type (N=16). The mice were 12-13 weeks old at the time of EV treatment. To administer EVs, mice were sedated using 2.5% (v/v) isoflurane/oxygen, and 2.25 × 10^7^ EVs in 50μl hibernation buffer were delivered into circulation via retro-orbital (RO) vein injection. Our previous published studies have demonstrated equivalent effectiveness utilizing either intra-hippocampal stereotaxic surgery or injection into the retro-orbital sinus (PMID: 33676561). EV treatments were given once a week for four weeks. Control AD and WT mice received RO injections on the same schedule using 50μl of vehicle.

**Cognitive Function Testing**

Behavioral testing began one month after EV treatment using N = 12-16 mice per group. The tests lasted for two weeks and included the following tasks: object recognition memory (ORM), elevated plus maze (EPM), and fear extinction (FE). All behavior videos were scored by independent investigators who were blinded to the experimental groups. ORM and EPM were scored manually, while FE was scored using FreezeFrame (Coulbourn Instruments).

**Object Recognition Memory (ORM)**

Object recognition memory measures episodic memory in which the animal’s preference for a novel object is measured. This task relies on the hippocampal, medial prefrontal cortex (mPFC), and perirhinal (PRh) cortex regions of the brain. The experimental setup includes testing rooms equipped with appropriate lighting (50 -70 Lux), four square arena boxes (30 x 30 x 30 cm), and camera recording hardware (Noldus). To habituate the mice to the ORM environment, mice explored the arena with bedding only for 3 days for 10 minutes. For the test day, the mice were presented with two plastic objects identical in color, shape, and size. Following a 5-minute exploration period, each mouse was returned to its home cage for another 5-minute phase. During this time, one familiar object was replaced with a novel object, and both objects were cleaned with 10% ethanol prior to the test phase. The mouse was then reintroduced to the arena for an additional 5 minutes of exploration. Each video was then manually scored by an individual blinded to the experiment. The discrimination index was calculated for each mouse using the formula: [(novel exploration time/total exploration time) – (familiar exploration time/total exploration time)] × 100.

**Elevated Plus Maze (EPM)**

The elevated plus maze is a measure of anxiety constructed of two bisecting platforms held together in a plus sign shape. One arm is open with no walls or roof, while the other is enclosed with walls on both sides, creating a darker environment. Before the task began, the platforms were cleaned with odor-free disinfectant (Virkon, University Laboratory Animal Resources). Then, each mouse was placed at the intersection of the platforms and given 5 minutes to explore. The same camera recording software (Noldus) was used to record the task. The time spent in the open arm versus the closed arm was measured and compared amongst each group to measure anxiety behavior. Again, each video was manually scored by an individual blinded to the experiment.

**Fear Extinction Memory**

We used a fear extinction memory task to determine if hNSCs or iMGL-derived extracellular vesicles in an AD brain affect amygdala-cortical-hippocampal circuit-dependent fear conditioning learning and memory consolidation. The fear extinction memory task reflects the functioning of the amygdala, mPFC and hippocampal circuit. The experimental setup consisted of a plexiglass box with a speaker connected to a sound system, a metal wire floor connected to an electric shock generator (ActiMetrics), and a plastic tray onto which an odor cue (10% vinegar) is sprayed. On day 1 of the task, a brief conditioning phase was conducted where the mice experienced three repeated sessions of an auditory stimulus paired with a subsequent mild foot shock. 24 hours later, the extinction training phase began and lasted for a total of 3 days. During this phase, the mice experienced 20 tones within the same behavioral chamber as the conditioning phase and the same odor. Twenty-four hours later, on the fifth and final day of the task, the fear test was conducted, exposing the animals to three tones within the same context as Day 1 but without any shock. The freezing behavior of the mice was recorded using a ceiling-mounted camera in each chamber and analyzed using an automated freezing measurement software (FreezeFrame, Coulbourn Instruments). The percentage of time each animal spent freezing was calculated for the conditioning, extinction, and test phases. Freezing times were averaged across 5-tone intervals for a total of 4 data points for each day of the extinction phase. Increased freezing during the FE task suggests an impaired fear memory consolidation process. Integrating data from these tests provides robust analytical tools for evaluating the effects of stem cell derived extracellular vesicles on cognitive function in an AD brain.

**Immunohistochemistry (IHC)**

After the behavioral tests were completed, the mice were euthanized via intracardiac perfusion with normal saline containing heparin (10 U/mL, Sigma) followed by 4% paraformaldehyde (PFA) in 100 mM PBS (pH 7.4, Sigma). Brains were then fixed overnight at 4°C in 4% PFA. Following this, tissues were cryoprotected using a 30% sucrose gradient in PBS supplemented with 0.02% sodium azide (Sigma). Cryosectioning was performed using a cryostat (HN525 NX, Epredia, Germany) at a coronal thickness of 30 µm. For each immunofluorescence stain, free-floating frozen brain sections were collected from each group for two distinct time points, which included 10 weeks post-EV injections and 4 months post-EV injections (2 sections per animal with visible mPFC and PRh regions, 4 brains per group). Thioflavin S staining was performed to visualize and quantify the amyloid plaques, by first washing the tissues with a gradient wash of ethanol (100%, 95%, 70%, 50%) for 3 minutes each (1). Subsequently, the tissues were incubated in 0.5% Thioflavin S (Sigma) solution in 50% ethanol for 10 minutes, protected from light. The tissues were then washed three times with 50% ethanol, followed by two washes with 1x PBS—first for 10 minutes and then for 5 minutes (Fiock, Betters, & Hefti, 2023). To quantify synaptic protein, the tissues underwent synaptophysin and PSD-95 immunofluorescence staining. The tissues were prepared by 3 washes of PBS (pH 7.4) for 5 minutes each, followed by a 30-minute blocking step with 4% bovine serum albumin (BSA) (Jackson ImmunoResearch) and 0.1% Triton X-100 (TTX, Sigma). The sections were then incubated for 24 hours in a primary antibody solution containing 2% BSA, 0.1% TTX, and mouse anti-synaptophysin (1:1000, Synaptic Systems) or Mouse anti PSD-95 (1:1000, Invitrogen). After primary antibody incubation, the sections were treated for 1 hour with goat anti-mouse IgG conjugated to Alexa Fluor 647 (1:1000). To observe the plaque targeted immune response, we utilized a plaque counterstain AmyloGlo (1:100 Biosensis) in conjunction with two different glial cell markers: the disease-associated microglia (DAM) protein CD9 (1:150, Biolegend), and glial fibrillary acidic protein (GFAP, 1:500, Sigma). In addition, dual immunofluorescence staining of complement C3d (goat anti-mouse C3d, 1:250, R&D Systems) also conducted in conjugation with GFAP. The tissues were first washed 3 times with 1X PBS for 5 minutes and then incubated in a 10% NGS + 0.1%TTX blocking solution, followed by a pre-stain with AmyloGlo, then overnight in the primary antibody. Fluorescence was visualized using goat anti-mouse Alexa Fluor 568 (1:500). To label activated microglia, IBA1-CD68 dual immunofluorescence staining was performed. The tissues were first washed with 0.3% Tween-20 (Sigma) 3 times for 5 minutes each. This was followed by a 3% hydrogen peroxide (Sigma) and 10% methanol (Sigma) treatment on ice. The tissues were then washed again with PBS 3 times for 5 minutes each and then incubated in a 4% BSA blocking solution and 0.3% PBS Tween-20 for 30 minutes at room temperature. Finally, the tissues were incubated overnight in primary antibodies (rabbit anti-IBA1, 1:500, Wako; and rat anti-mouse CD68, 1:500, BioRad). Fluorescence was visualized using goat anti-rat Alexa Fluor 647 (1:1000) and goat anti-rabbit Alexa Fluor 488 (1:500) secondary antibodies.

**Microscopy and 3D Algorithm-based Volumetric Quantification**

Single (Synaptophysin and Thioflavin S) and Dual (CD68-IBA1, C3d-GFAP, GFAP-AmlyoGlo, and CD9-AmyloGlo) immunofluorescence-stained sections were imaged using a Nikon Eclipse AX laser scanning microscope (Nikon Eclipse AX, Japan) equipped with either a 20x air or 40x oil immersion objective lens. High-resolution images (1024 to 2048p) with z stacks (0.5 to 1 µm thick) were acquired using the NIS Element AR module (v4.3, Nikon). Images range from 15-25 µm thick. These images were then deconvoluted using 3D volumetric analysis software (ClearView, Imaris v9.2, BitPlane, Inc.). For Thioflavin S, the plaques were counted from each image taken from the confocal microscope. For synaptophysin, the surface volume of the synaptic puncta was obtained and analyzed. For CD68-IBA1 and C3d-GFAP, the volumetric co-localization between the surfaces of the two markers for each stain was measured and analyzed. For GFAP-AmyloGlo and CD9-AmyloGlo, quantification of total immunoreactivity of both CD9 and GFAP were analyzed, in addition to the colocalized immunofluorescent expression of either CD9 or GFAP with AmyloGlo; the same individual and colocalized quantification was performed for the C3d-GFAP staining.

**Transcriptomic Profile of Neuroinflammation in Alzheimer’s Mouse Brain Treated with EVs**

To determine the therapeutic effects hNSC- and iMGL-derived EV treatment on AD brain neuroinflammation gene markers, samples from 18 mice, ages 25-26 weeks at the time of perfusion, were analyzed using the commercially available nCounter® Mouse Neuroinflammation Panel from NanoString™, which categorizes the expression of 757 genes related to neuroinflammation. Total RNA was isolated from 100 mg of fresh-frozen brain tissue per sample, with 4-5 samples for each of the 4 treatment groups. Samples were first homogenized in QIAzol Lysis Reagent (Qiagen) and then underwent RNA isolation with RNeasy Plus Universal Mini Kit (Qiagen) to obtain minimum of 100ng of unreplicated RNA, whose purity was verified with both NanoDrop (ThermoFisher) and Bioanalyzer 2100 (Agilent Technologies). 5 µl of total RNA (20 ng/µl) per sample were labeled with a fluorescent Reporter ProbeSet, hybridized, and analyzed per the manufacturer’s instructions: NanoString™ (Seattle, WA). The Genomics Research and Technology Hub at UC Irvine carried out the data collection. The raw value data were normalized on the nCounter Software utilizing a two-step normalization process: an initial Positive Control Normalization, followed by a CodeSet Content Normalization using 13 internalized housekeeping genes.

**RNA-seq of Unique EV miRNA Cargo**

MicroRNAs (miR) are key bioactive cargo within EVs that exert varied and long-reaching effects on intra- and inter-neuronal activity (PMID: 30252569). To better characterize and compare the unique miR signature of hNSC- and iMGL-derived EVs, pure RNA was isolated from EV stock solutions using the RNEasy Kit (Qiagen); nucleic purity was verified with both NanoDrop (ThermoFisher) and Bioanalyzer 2100 (Agilent Technologies). Isolated RNA from both EVs was then sequenced using the NEXTFLEX Small RNA Sequencing Kit v4 (Revvity), with sample processing and data organization conducted by the Genomics Research and Technology Hub at UC Irvine. For each of the two unique miR library sets, we implemented selection criteria to include miR whose gene counts constituted more than 1% of total miR expression or were expressed in both library sets.

**References for the Supplemental Methods:**

Apodaca, L. A., Baddour, A. A. D., Garcia, C., Jr., Alikhani, L., Giedzinski, E., Ru, N., . . . Baulch, J. E. (2021). Human neural stem cell-derived extracellular vesicles mitigate hallmarks of Alzheimer's disease. *Alzheimers Res Ther, 13*(1), 57. doi:10.1186/s13195-021-00791-x

Fiock, K. L., Betters, R. K., & Hefti, M. M. (2023). Thioflavin S Staining and Amyloid Formation Are Unique to Mixed Tauopathies. *J Histochem Cytochem, 71*(2), 73-86. doi:10.1369/00221554231158428

McQuade, A., Coburn, M., Tu, C. H., Hasselmann, J., Davtyan, H., & Blurton-Jones, M. (2018). Development and validation of a simplified method to generate human microglia from pluripotent stem cells. *Mol Neurodegener, 13*(1), 67. doi:10.1186/s13024-018-0297-x
